# The 2018 biomembrane curvature and remodeling roadmap

**DOI:** 10.1088/1361-6463/aacb98

**Published:** 2018-07-20

**Authors:** Patricia Bassereau, Rui Jin, Tobias Baumgart, Markus Deserno, Rumiana Dimova, Vadim A Frolov, Pavel V Bashkirov, Helmut Grubmüller, Reinhard Jahn, H Jelger Risselada, Ludger Johannes, Michael M Kozlov, Reinhard Lipowsky, Thomas J Pucadyil, Wade F Zeno, Jeanne C Stachowiak, Dimitrios Stamou, Artú Breuer, Line Lauritsen, Camille Simon, Cécile Sykes, Gregory A Voth, Thomas R Weikl

**Affiliations:** 1 Laboratoire Physico Chimie Curie, Institut Curie, PSL Research University, CNRS UMR168, 75005 Paris, France; 2 Sorbonne Université, 75005 Paris, France; 3 Chemistry Department, University of Pennsylvania, Philadelphia, PA 19104-6323, United States of America; 4 Department of Physics, Carnegie Mellon University, Pittsburgh, PA 15213, United States of America; 5 Department of Theory and Bio-Systems, Max Planck Institute of Colloids and Interfaces, Science Park Golm, 14424 Potsdam, Germany; 6 Biofisika Institute (CSIC, UPV/EHU) and Department of Biochemistry and Molecular Biology, University of the Basque Country, Leioa 48940, Spain; 7 IKERBASQUE, Basque Foundation for Science, Bilbao 48013, Spain; 8 Federal Research and Clinical Centre of Physical-Chemical Medicine, Moscow 119435, Russia; 9 A.N. Frumkin Institute of Physical Chemistry and Electrochemistry, Russian Academy of Sciences, Moscow 119071, Russia; 10 Department of Theoretical and Computational Biophysics, Max Planck Institute for Biophysical Chemistry, Göttingen, Germany; 11 Department of Neurobiology, Max Planck Institute for Biophysical Chemistry, Göttingen, Germany; 12 Department of Theoretical Physics, Georg-August University, Göttingen, Germany; 13 Cellular and Chemical Biology Unit, Institut Curie, PSL Research University, U1143 INSERM, UMR3666 CNRS, 26 rue d’Ulm, 75248 Paris Cedex 05, France; 14 Sackler Faculty of Medicine, Department of Physiology and Pharmacology, Tel Aviv University; 15 Indian Institute of Science Education and Research, Pune, India; 16 Department of Biomedical Engineering, University of Texas at Austin, Austin, TX, United States of America; 17 University of Texas at Austin, Institute for Cellular and Molecular Biology, Austin, TX, United States of America; 18 Bionanotechnology and Nanomedicine Laboratory, Department of Chemistry, Nano-Science Center, University of Copenhagen, Denmark; 19 Department of Chemistry, James Franck Institute, and Institute for Biophysical Dynamics, The University of Chicago, Chicago, IL, United States of America

**Keywords:** biomembrane, curavture, remodeling

## Abstract

The importance of curvature as a structural feature of biological membranes has been recognized for many years and has fascinated scientists from a wide range of different backgrounds. On the one hand, changes in membrane morphology are involved in a plethora of phenomena involving the plasma membrane of eukaryotic cells, including endo- and exocytosis, phagocytosis and filopodia formation. On the other hand, a multitude of intracellular processes at the level of organelles rely on generation, modulation, and maintenance of membrane curvature to maintain the organelle shape and functionality. The contribution of biophysicists and biologists is essential for shedding light on the mechanistic understanding and quantification of these processes.

Given the vast complexity of phenomena and mechanisms involved in the coupling between membrane shape and function, it is not always clear in what direction to advance to eventually arrive at an exhaustive understanding of this important research area. The 2018 Biomembrane Curvature and Remodeling Roadmap of *Journal of Physics D: Applied Physics* addresses this need for clarity and is intended to provide guidance both for students who have just entered the field as well as established scientists who would like to improve their orientation within this fascinating area.

## Membrane curvature and BAR-domain proteins

Patricia Bassereau^1,2^

^1^ Laboratoire Physico Chimie Curie, Institut Curie, PSL Research University, CNRS UMR168, 75005 Paris, France

^2^ Sorbonne Université, 75005 Paris, France

### Status.

**Figure 1.**
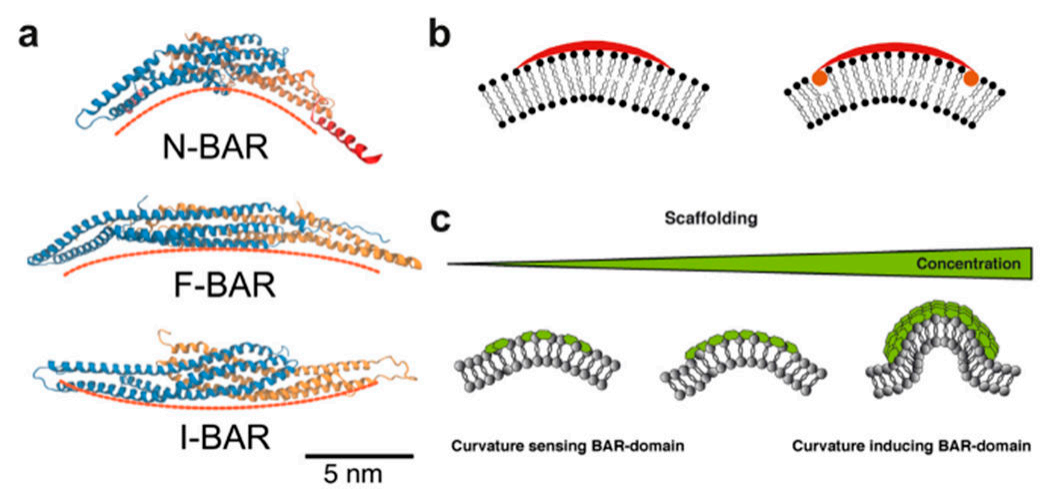
Some aspects of BAR-domain proteins on membranes. (a) Three characteristic types of BAR domain proteins with different intrinsic curvatures, positive (N-BAR, F-BAR) or negative (I-BAR). Reprinted from [1], Copyright 2015, with permission from Elsevier. (b) When they bind on one side of a negatively charged lipid membrane, they induce membrane bending. This deformation can also be amplified by amphipathic helices insertion (right), as in the case of the N-BAR domains. Reprinted from [3], Copyright 2013, with permission from Elsevier. (c) Different regimes exist, depending on the protein density on the membrane, from curvature sensing to curvature generation. Reproduced with permission from [4]. Copyright © 2014 Cold Spring.

Cell membranes are highly curved during key cellular processes, such as membrane trafficking, cytokinesis, infection, immune response, or cell motion. Proteins with Bin/amphiphysin/Rvs (BAR) domains with intrinsically curved and anisotropic shapes (figure 1(a)) have been shown to be essential in many of these processes. When their unique structure was uncovered, their capability to locally deform membranes was also demonstrated [2] (figure 1(b)). However, it took about a decade to build up a comprehensive modeling of their mechanical modes of action on membranes. During this period, different *in vitro* assays have been developed using model membranes and purified BAR-domains, coupled to theoretical models based on thermodynamics and spontaneous curvature. Generally, two regimes can be distinguished depending on the actual surface fraction of proteins on the membrane (figure 1(c)): at low density, BAR domains are essentially curvature-sensors with a ‘sensing strength’ depending on their intrinsic curvature but at high density, they induce curvature [1]. In this last regime, they form scaffolds that mechanically constrain membrane tubes or bud necks and spontaneously tubulate membranes [5]. Depending on the proteins, these scaffolds can result only from self-assembling in the absence of protein–protein interactions [6] or from direct protein-protein interactions [7]. In the case of protein with shallow curvature (see I-BAR figure 1(a)), phase-separation between low- and high-density phases occurs at low curvature [8]. Eventually, friction between BAR scaffolds and membrane leads to tube scission when an elongation force is applied [9]. Coarse-grained (CG) simulations have also been very influential for the field, showing how the proteins assemble at supramolecular scale and the consequences on the membrane shape [1, 10].

Globally, understanding has progressed and a general framework of BAR-domain functioning has emerged, but there are still some dark zones and open questions unsolved. For instance, striking differences have been reported for BAR domains interacting either with isolated spheres or with spherical buds or tubes connected to a flat membrane, which are not fully addressed with current models. More questions will be developed in the following sections. Nevertheless, since BAR-domain proteins are found associated to more and more cellular functions all over in cells, more accurately understanding their action depending on their molecular structure and their interactions with other cell components are a new challenge for the coming years.

### Current and future challenges.

#### Advanced models including anisotropic curvature and molecular details.

Globally, mechanics-based methods for *in vitro* assays in their current form capture the essentials of membrane shaping by BAR domains, but fail to distinguish the structural details. So far, although some theoretical models include an anisotropic spontaneous curvature for the BAR domains, only an isotropic spontaneous curvature and simple steric interactions between proteins have been introduced in the current models used for the analysis of tube pulling or spontaneous tubulation experiments. This description is too limited and cannot account for the discrepancy between the spontaneous curvature values deduced from different methods (e.g. tube assay or spontaneous tubulation), the absence of tubulation for some BAR-domain proteins, or the correlation between intrinsic curvature and facility for tubulation. CG simulations have predicted a rich variety of behaviours when including the protein anisotropic curvature (different curvatures between the BAR backbone and the lateral direction) [10]. Thus, the next challenge is to develop more comprehensive theoretical models that better integrate molecular and structural details, protein–protein and protein–membrane interactions, especially the mean and Gaussian curvature contributions, as well as dynamical aspects. Parameters for such a model might be calculated from a CG simulation of the protein on a membrane, but this also requires progresses in cross-scale simulations.

#### Effect of BAR-domains on lipids.

The origin of phosphoinositide clusters: BAR and I-BAR domains have been shown to induce a local clustering of PiP2 lipids, larger than the number of lipids expected to be bound considering the protein charges. To my knowledge, the detailed mechanism behind this effect is not understood although this clustering certainly has important biological consequences.

#### BAR-domains and diffusion barrier.

BAR domain scaffolds strongly limit the diffusion of non-charged lipids underneath as well as their advection when a membrane flow is produced. So far, the published data suggest that this effect exists on both leaflets even when the BAR domain is bound only on one. But more experiments would be required to explore this question as well as a model for a deeper understanding of the origin of the diffusion barrier.

#### Physics of membrane budding and protruding.

Clathrin-mediated endocytosis in mammalian cells is accompanied by the sequential binding of BAR domain proteins of increasing intrinsic curvature, as well as by the concomitant recruitment and transformation of different phosphoinositides (a process named ‘curvature cascade amplification’ by Gallop). At the same time, actin polymerisation is initiated around the growing bud that participates to the budding process. Similarly, when filopodial protrusions are formed, a sequence of I-BAR domain recruitment, actin nucleators and finally actin polymerisation takes place. So far, a full physical model and *in vitro* reconstitution of these processes are still missing that would allow to couple the change in membrane shape to BAR-domain recruitment, phosphoinositide clustering and transformation, and finally to actin growth and force production.

### Advances in science and technology to meet challenges.

#### Progress on in vitro assays.

The current single nanotube assays (developed in Bassereau’s and Baumgart’s groups) are quite accurate for precise quantification of the interaction between BAR domains and membranes but lack parallelization. In contrast, the new high-throughput platform from Pucadyil (supported membrane tubes (SMrT)) [11] allows for parallel measurements but suffers from contacts between nanotubes and substrate. With the single liposome curvature assay (CLiC) designed by Stamou [12], hundreds of small liposomes can be studied simultaneously, but since liposomes are tethered to a solid substrate, exchange with a flat membrane reservoir cannot be considered as well as questions related to bud neck geometry. Considering the I-BAR domain proteins, they require to be encapsulated inside liposomes for probing their affinity with negative curvature, but current methods have their limitation. Moreover, no model system is available with a controlled negative Gaussian curvature. Thus, there is a clear need for the design and engineering of novel *in vitro* systems with controlled geometry that mimic cellular situations, allowing for quantitative imaging and possibly mechanics and high-throughput. At the same time, as mentioned in the above section, more advanced theoretical models must be set up for a comprehensive analysis of these experiments.

#### In vivo quantification.

Since *in vitro* experiments predict different behaviours for BAR-domains that depend on their density on membrane, the actual densities on biological membranes must be measured. Recent developments in cell biology with controlled expression levels of fluorescent proteins (CRISPR/Cas9) together with super resolution microscopy and 3D imaging (e.g. light sheet microscopy) should allow quantification in the future of the number and the densities of BAR-proteins involved in different cellular processes, and thus bridging the *in vivo* and the physical models. On the same line, these tools should be used to image and measure the clustering effect of BAR-domains on phosphoinositide lipids (such as Pi(4,5)P2) with a time and spatial resolution good enough to establish the role of lipid clustering in BAR-related budding or protruding events.

#### Concluding remarks.

This short review on the current and future challenges related to BAR domains probably reflects my own interests and certainly misses important issues. New tools have to be developed to allow bridging the gap between molecular and structural knowledge and microscopic and mechanical descriptions. This will also require new technical developments *in vitro* and *in vivo*. BAR domains with their unique shape and properties still represent a puzzling problem since they build localized platforms coupling membrane mechanics (curvature), biochemistry (PiP2) and force production (actin polymerisation), thus remaining interesting for cell biologists, computational scientists and biophysicists.

Acknowledgments

I thank all former collaborators who contributed to the work on BAR-domains proteins that inspired this paper. I acknowledge the support of the Agence Nationale pour la Recherche and of Human Frontier Science Program Organization. The PB group belongs to the CNRS consortium CellTiss and to Labex CelTisPhyBio (ANR-11-LABX0038) and to Paris Sciences et Lettres (ANR-10-IDEX-0001-02).

## Amphipathic helices coupling with membrane curvature

Rui Jin and Tobias Baumgart

Chemistry Department, University of Pennsylvania, Philadelphia, PA 19104-6323, United States of America

### Status.

Amphipathic helices (AHs) considered in this contribution consist of a polypeptide-helix with opposing polar and nonpolar faces oriented along the central axis. This type of AH is a common motif found in membraneremodeling proteins and peptides.

In most cases, AHs are unfolded in an aqueous environment. The transition from a disordered to an AH conformation occurs upon membrane binding, at which point the hydrophobic face of the AH is buried within the hydrophobic membrane interior and the polar face is exposed to the hydrophilic lipid head groups and aqueous phase. The membrane-inserted AH may facilitate the generation of curved membrane structures, including vesicles, tubules and even membranes with saddle shape. Many AHs contain charged residues on the polar face, which complicates AH-membrane interactions and further contributes to the diverse membrane remodeling phenomena induced by AHs.

Segrest *et al* grouped AH-containing proteins into several classes according to their functions. The main differences were found in the net charge and charge distribution, as well as the angle *a* spanned by the polar face [13]. Later studies of specific proteins provided closer links between the mechanisms of their function and properties of their AH motifs. For example, one subgroup of antimicrobial AH peptides are relatively short with net positive charge ranging from +2 to +9. Their *in vivo* role is to kill bacteria by deforming membranes and creating membrane pores [14]. N-BAR-domain containing proteins bear a short, charged N-terminal AH. This helix anchors the protein to the plasma membrane to facilitate endocytosis. Another plasma membrane-remodeling protein, alpha-synuclein, has a uniquely long AH structure at the N-terminal side of the protein with positive charges distributed over the polar/nonpolar interface, and this protein is involved in complex membrane reshaping events. In contrast, the AHs in the curvature generating proteins Sarlp and Arf1 are more hydrophobic with lower net charge, and these AHs are recruited to ER and Golgi membranes [15].

*In vitro* studies based on a variety of different experimental techniques have served to investigate the membrane shape-dependent binding behavior and helix formation of AHs. Lipid tethers of different radii can be pulled from giant vesicles (with radii on the order of tens of *μ*m). Numerous AHs show increased surface density on the membrane when the tether radius decreases, i.e. they show ‘curvature sensing’ [16]. Curvature sensing can also be characterized by measuring AH density on single liposomes of difference curvatures (SLiC assay). Both NMR and ESR experiments can be used to verify helix formation, and fluorescence quenching experiments can assess membrane insertion [17]. Oriented circular dichroism can further provide information about helicity and orientation relative to the membrane plane [18].

**Figure 2.**
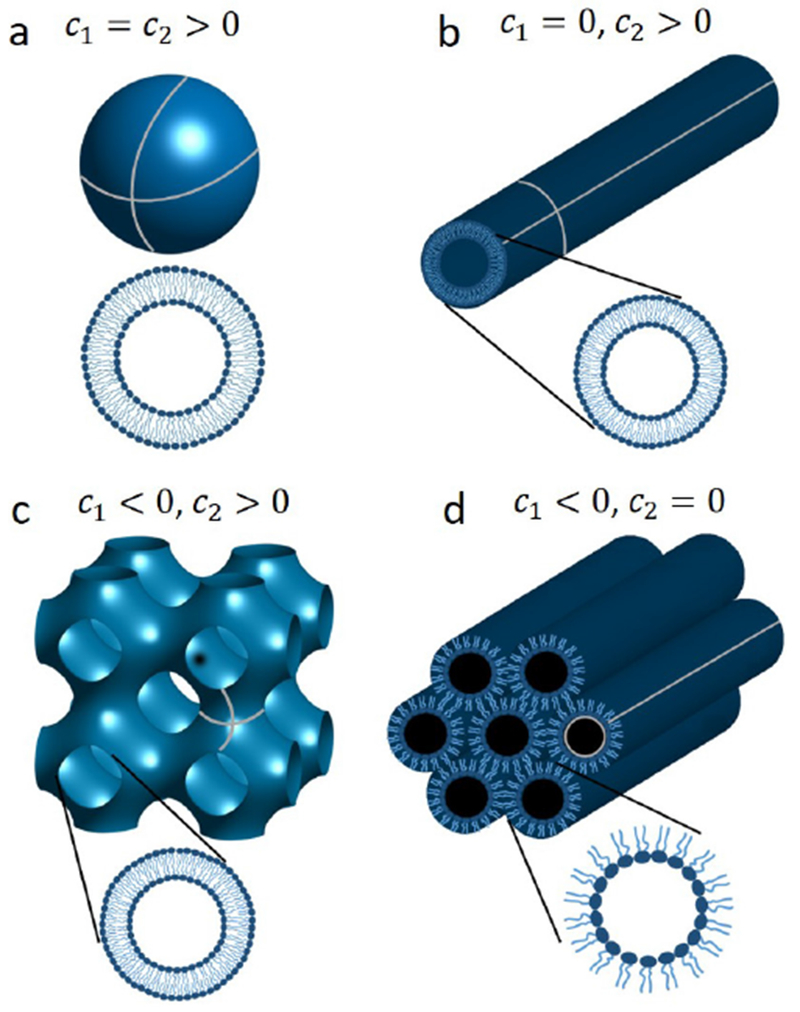
Types of membrane geometries with different mean and Gaussian curvatures. (a) A vesicle with positive mean curvature and positive Gaussian curvature. (b) A tubule with positive mean curvature and zero Gaussian curvature. (c) The cubic phase lm3m with negative Gaussian curvature. (d) The inverted hexagonal phase HII with negative mean curvature and zero Gaussian curvature. The principal directions are shown in grey.

*In vitro* studies have also been used to assess membrane deformation. When AH-containing proteins bind to the lipid membrane, shape changes can be directly observed as vesiculation and tubulation through electron or fluorescence microscopy imaging [16] (figures 2(a) and (b)). X-ray diffraction has been frequently applied to detect structural membrane phases related to negative curvature (figures 2(c) and (d)) [19].

### Current challenges and future directions.

As discussed above, with similar structures, AHs may show distinct membrane interaction behavior and induce different forms of membrane deformation. In general, the principal curvatures (*c*_1_ = 1/*R*_*max*_, *c*_2_ = 1/*R*_*min*_), which are measured along orthogonal directions on the membrane surface, may be positive, negative, or zero if one or both of these lines are straight. Both mean curvature *H* = (*c*_1_ + *c*_2_)/2 and Gaussian curvature *K* = *c*_1_*c*_2_ are needed to describe the membrane geometry. For vesicles, both the Gaussian and the mean curvatures are non-zero (figure 2(a)), while cylindrical tubules have zero Gaussian curvature and a non-zero mean curvature (figure 2(b)). The diversity of membrane geometries complicates the analysis of AH function. Specific analytical models may only be applicable to specific membrane deformation modes, as we now discuss.

**Figure 3.**
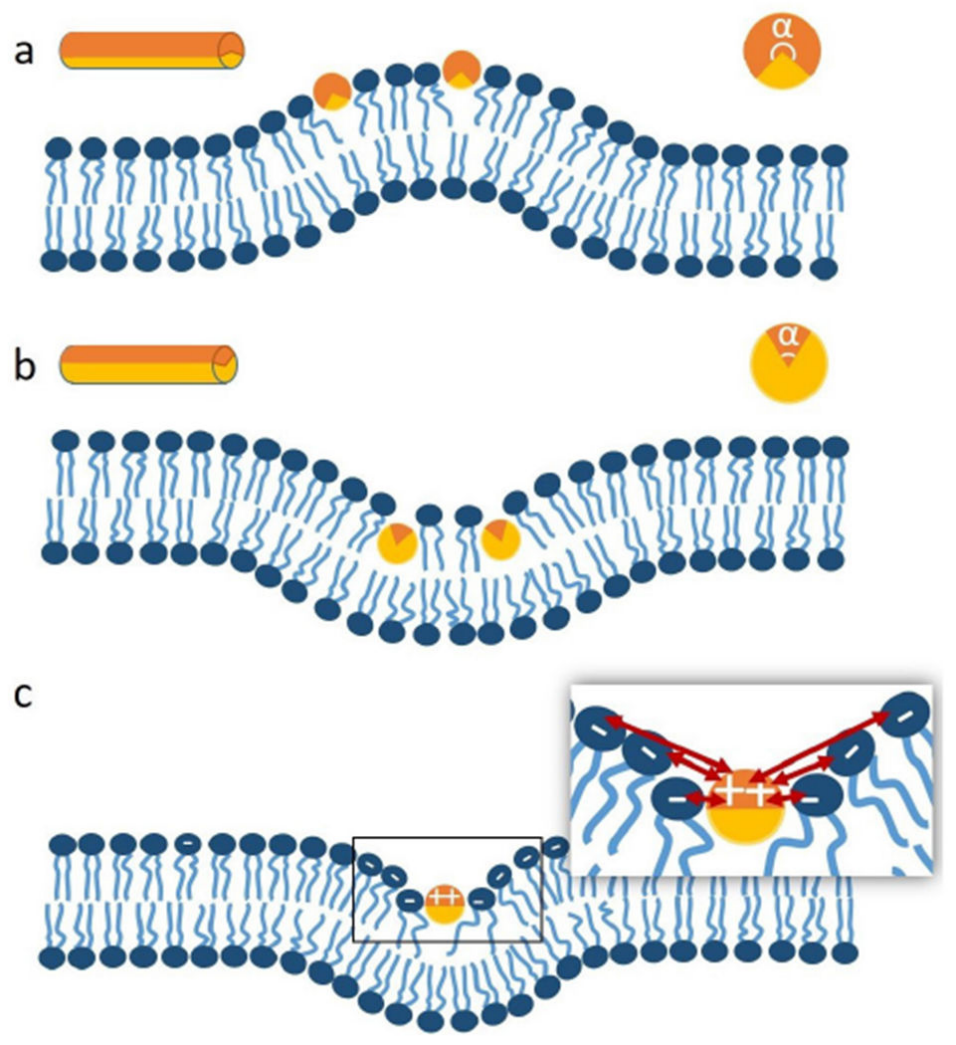
Mechanistic models of AH-induced membrane curvature. (a) Helix insertion/wedging induces positive membrane curvature and the hydrophobicity distribution on the helix. Orange color indicates the hydrophilic region (spanning polar angle *α*) while yellow denotes the hydrophobic region. (b) Deeper insertion of AHs induces negative curvature. (c) Negative curvature induced by electrostatic wrapping. The red arrows represent electrostatic interactions.

In mechanistic models, AHs have been treated as rod-like cylinder inclusions, and the lipid bilayer was considered as an anisotropic elastic material. The helix insertion model claims that the AH locally expands the bilayer and produces intramembrane stress. Membrane curving from the initial flat state is generated to minimize the elastic energy of the membrane (figure 3). While shallow insertion of a helical peptide with the long axis parallel to the membrane surface can induce positive membrane curvature (i.e. away from the peptide), deeper insertion may induce membrane curvature in the opposite direction (negative curvature) [14, 20]. The polar angle of the AH (see figure 3) and the AH size (such as length and width) may contribute to the insertion depth. Associating these properties with membrane curvature generation is still under investigation.

Another possible mechanism for negative curvature generation may be electrostatic wrapping of cationic peptides by membranes containing anionic lipids (figure 3(c)) [21]. The competition between the electrostatic and the curvature elastic contribution to the system’s free energy determines if a concave membrane shape is favored. The question as to what extent hydrophobic insertion versus electrostatic wrapping contributes to the function of specific peptides has thus far remained unanswered. Moreover, for membranes with negative Gaussian curvature, positive and negative curvatures exist in different directions (figure 2(c)). There currently are no quantitative models describing such a complex situation. In addition to generic hydrophobic and electrostatic interactions, it is likely that specific hydrogen bonding interactions have to be considered to explain negative Gaussian curvature generation. Furthermore, all existing mechanistic models are based on the assumption that the AH lies flat on the membrane surface. Particular lipid compositions and interactions with other types of macromolecules within the membrane may induce a tilted and even perpendicular orientation of the AH relative to the membrane surface. Such varied insertion angles may be an interesting target for both theoretical study and experimental investigation [22].

While the mechanistic models could help in acquiring a broad understanding of AH–membrane interactions, MD simulations can help to reveal more interaction details. Simulations can be used to define the free energy landscape for AH formation and its insertion into a lipid bilayer, as well as investigating the preferred insertion depth and orientation. However, there is still a long way to go to establish the relationship between a peptide sequence and its preferred conformation upon contact with lipid membranes. Further attention should be paid to account for effects of the local peptide environment, including pH, lipid composition, and macromolecules which cooperate in deforming membranes or induce environmental variations. Moreover, the large majority of MD simulations of AH/membrane interactions are based on the use of non-polarizable force fields. However, it is likely that the rapidly changing dielectric environment across the lipid bilayer requires the use of polarizable force fields to accurately describe electrostatic interactions between charged peptides and membranes. A polarizable force field based on a simple Drude oscillation model has been proposed and may lead to improved MD simulations of AH/membrane interactions [23].

For experimental studies, the investigation of negative mean curvature sensing is more challenging compared to that of positive curvature as it may require encapsulation of the peptide within a closed membrane [16]. Negative Gaussian curvature (i.e. saddle shape) generation accompanies membrane processes such as fusion and fission. However, contrary to positive curvature generation, it is challenging to directly quantify. Consequently, indirect approaches have prevailed. These include the detection of phase transition temperature modulations through negative membrane curvature generators, in transitions from lamellar to hexagonal phase (figure 2(d), negative mean curvature) and lamellar to cubic phase (figure 2(c), negative Gaussian curvature). These studies are often complemented by x-ray diffraction to verify and characterize the resulting membrane structures [19]. While such studies have often been carried out in DOPE membranes, it is increasingly clear that lipid composition can play a major role in modulating the function of the peptide, and even reverse the sign of curvature generation [24]! Clearly, this aspect warrants more attention. Furthermore, it is not straightforward to relate cubic and hexagonal phase morphologies with membrane geometries found *in vivo.*

### Concluding remarks.

Although the structure of amphipathic helices appears deceptively simple, AHs engage in several different modes of membrane curvature generation. To gain deeper insight into their mechanism of function, we need to further clarify the differences and similarities between the interactions of membranes with different types of AHs. More attention should be paid to the effects of the local environment on the function of AHs on membranes. The results of such endeavors will clarify the physiological role of naturally occurring peptides and guide the design of synthetic peptides for therapeutic applications.

Acknowledgments

We thank Samantha Wilner and Jaclyn Robustelli for discussions and acknowledge funding from NIH Grant R01 GM 097552.

## Gaussian curvature, membrane topology, and the energetics of membrane fusion

Markus Deserno

Department of Physics, Carnegie Mellon University, Pittsburgh, PA 15213, United States of America

### Status.

The Gaussian contribution to Helfrich’s classical curvature-elastic energy does not depend on details of a membrane’s shape. More precisely, the Gauss–Bonnet theorem states that

(1) $$\int_{M}{\mathrm{dAK}}_{G}+\oint_{\partial M}{\mathrm{dAk}}_{g}=2\pi \chi (M),$$

where K_G_ is the Gaussian curvature and k_g_ the geodesic curvature at the boundary ∂M of a surface M with Euler characteristic *χ*(M). Hence, neither the Gaussian energy nor its associated modulus $\bar{\kappa }$ matter for membrane energetics, unless there is a change in boundary (this includes contact lines between two membrane phases of differing $\bar{\kappa }$) or topology. For the latter case, fission and fusion events (Δ_*χ*_ = ±2) are the most important examples.

**Figure 4.**
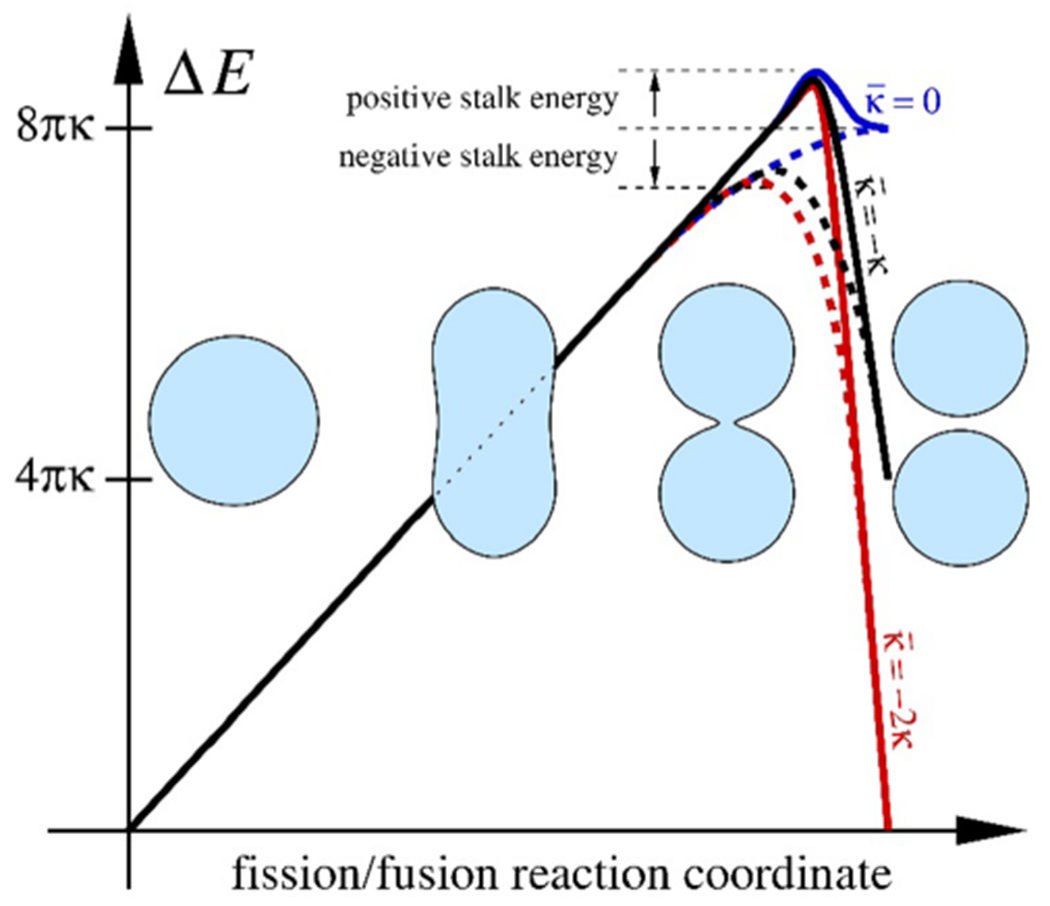
Simplified schematic of the energy change Δ*E* for a spherical vesicle splitting into two. Up to shortly before fission, a total energy of approximately *8πκ* of ordinary bending energy accumulates, followed by a topological term $4\pi \bar{\kappa }$ < 0 upon scission. The non-bilayer intermediate stalk conformation can increase or lower the energy at the transition state, depending on the lipid composition, especially the spontaneous monolayer curvature [26].

As illustrated in figure 4, reshaping a spherical vesicle into two spheres joined by a narrow neck incurs only ordinary bending energy (approximately 8*πκ* ≈ 500 k_B_T; the catenoidal neck is a minimal surface and therefore does not contribute). Cells accomplish this step with proteins that progressively remodel some patches of a membrane into a nascent bud (as discussed in the sections of Bassereau (section 1), Jin and Baumgart (section 2), Kozlov (section 8), Zeno and Stachowiak (section 11), Simon and Sykes (section 13), and Voth (section 14) in this Roadmap). Subsequent fission (usually by dynamin, see the sections by Frolov and Bashkirov (section 5) and Pucadyil (section 10) in this Roadmap) lowers the energy by the ‘instant’ topological contribution $4\pi \bar{\kappa }<0$, and unless $|\bar{\kappa }∕\kappa |\ll 1$, this is comparable in magnitude to the initial bending term. The latter follows because $\bar{\kappa }∕\kappa \in [-2,0]$ is the permissible range within which Helfrich’s curvature energy density is positive definite and hence a lamellar phase is stable [25].

Unlike for fission, the barrier for the reverse process of fusion is therefore topological. Overcoming it requires re-connecting two individual leaflets, which have their own elastic monolayer moduli *κ*_m_ and ${\bar{\kappa }}_{m}$, as well as a spontaneous monolayer curvature K_0,m_. For sufficiently negative K_0,m_, and after also accounting for lipid tilt, the half-way intermediate stalk structure can be energetically favorable by several tens of k_B_T [26], but the topological barrier $|4\pi \bar{\kappa }|$ could be even larger than that. By its very nature, it cannot be climbed continuously, rendering fusion a fundamentally more challenging process to orchestrate than fission (a specific example is discussed in the section of Grubmüller *et al* (section 6) in this Roadmap). This suggests that, irrespective of the functional details of a protein-based fusion machinery, cells should have a strong incentive to decrease $|\bar{\kappa }|$, at least locally. Indeed, the monolayer-bilayer consistency relation $\bar{\kappa }=2({\bar{\kappa }}_{m}-2\kappa_{m}z_{0}K_{0,m})$ implies that a more strongly negative spontaneous monolayer curvature reduces not only a stalk’s bending energy [26] but the overall topological barrier $|4\pi \bar{\kappa }|$ (here, z_0_ > 0 is a monolayer’s pivotal plane position). Unfortunately, it is difficult to know the absolute numbers, because the Gauss–Bonnet theorem makes it hard to measure the Gaussian modulus (a few exceptions are listed in table 1 of [27]).

### Current and future challenges.

Membrane remodeling is a crucial prerequisite for trafficking in eukaryotic cells, and considering how strongly its energetics may be affected by the Gaussian curvature modulus, it is disconcerting how little we still know about this elastic parameter. Overcoming the Gauss–Bonnet theorem is technically challenging, because it is difficult to either work with open membrane edges or change membrane topology in a sufficiently controlled way. In a situation like this, a possible way forward is additional modeling: constructing a finer-scale explanatory framework for the larger-scale curvature-elastic theory and its otherwise empirical moduli. Indeed, very general continuum elastic considerations suggest that the Gaussian curvature modulus $\bar{\kappa }$ and its monolayer counterpart ${\bar{\kappa }}_{m}$ can be expressed as the second moment of a membrane’s lateral stress profile *σ*_0_(z), taken over the bilayer or (when centered at the pivotal plane z_0_) a monolayer leaflet, respectively [28, 29]:

(2) $$\bar{\kappa }=\int_{-d∕2}^{d∕2}\mathrm{dz}\;z^{2}\sigma_{0}(z),\;\;{\bar{\kappa }}_{m}=\int_{0}^{d∕2}\mathrm{dz}{\left(z-z_{0}\right)}^{2}\sigma_{0}(z).$$

We do not yet know how to measure the stress profile in experiments, but it is readily accessible in simulations and has indeed been used to calculate these moduli. Unfortunately, the results are at odds with an alternative direct method that monitors curved open-edge membranes [27, 30], and equation (2) often yields $\bar{\kappa }>0$ [30, 31], outside the permissible stability range. This has been very puzzling, because it is unclear where a discrepancy could even arise.

Furthermore, real biomembranes are complex mixtures, whose elastic parameters will depend on the specific lipid composition. Even for the ‘easy’ parameters and simple binary mixtures this may yield unexpected behavior: for instance, neither the ordinary rigidity *κ* [32] nor the spontaneous monolayer curvature K_0,m_ [33] are simple linear (‘lever rule’) combinations of the values they take in pure phases. In other words, it is generally incorrect to assume that elastic parameters can be attributed to a single lipid irrespective of its environment. We must hence assume that this non-additivity also holds for the Gaussian curvature modulus, leaving us at the moment with no reliable predictions of $\bar{\kappa }$ for any biologically realistic membrane.

### Advances in science and technology to meet challenges.

Making progress with Gaussian moduli will require a concerted effort of experiment, theory, and simulation. Experimentally, new protocols should be explored that address the Gauss–Bonnet constraint by explicitly monitoring open boundaries or topology changes. Specifically, it will be important to allow for the case that $\bar{\kappa }∕\kappa$ is not close to zero (and hence neither spontaneous fusion nor a change into a non-lamellar phase is energetically easy, as $|4\pi \bar{\kappa }|\gg k_{B}T$), because we do not yet know what value of $\bar{\kappa }∕\kappa$ is biologically relevant. Moreover, the case of mixtures will invariably raise the issue of composition-curvature coupling—not just for the Gaussian moduli: any curvature gradients will trigger a redistribution of lipids (and vice versa). This not only renders the shape equations much harder to solve, but also introduces new coupling constants that must ultimately be linked to some underlying lipid physics.

**Figure 5.**
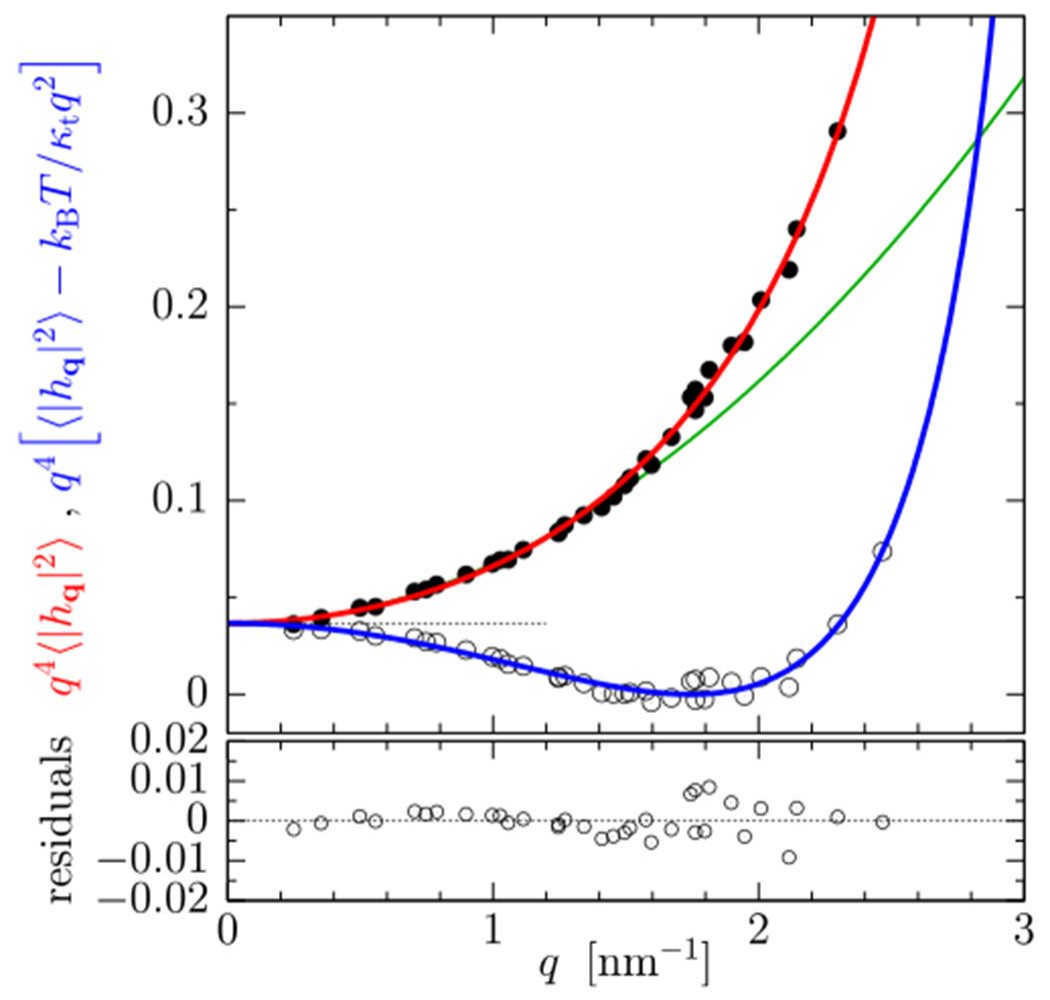
Power spectrum of membrane undulations for the CG MARTINI model of DPPC at 50 °C. Reprinted with permission from [34]. Copyright 2017, AIP Publishing LLC. The red and blue curves are fits to a revised theory including a new tilt-curvature coupling term from which ${\bar{\kappa }}_{m}$ can be extracted; the green curve is a fit using the original theory [29] (using wave vectors up to *q* = 1.5 nm^−1^).

On the theory side, the reason why the stress profile moments in equation (2) fail to predict the Gaussian moduli needs to be found, because the underlying continuum elastic framework [29] is very valuable: it offers a powerful predictive route to the macroscopic physics and severely reduces the number of independent symmetry-permitted parameters, while making remarkably minimalist modeling assumptions. It has recently been claimed that this theory should indeed be amended by an additional term that couples tilt with curvature at the relevant quadratic order [34], but this correction does not resolve the stress profile issue (equation (2) is still found to be true). However, [34] offers an unexpected new access to the microphysics, because the prefactor of the novel coupling turns out to be the second moment of a monolayer’s stress profile, which equals ${\bar{\kappa }}_{m}$ in this context. This liberates ${\bar{\kappa }}_{m}$ from the shackles of Gauss–Bonnet and permits it to enter shape equations and fluctuation expressions. For instance, it leads to significant changes in the power spectrum $\langle {|h_{q}|}^{2}\rangle$ of membrane undulations at large wave vectors (q ≳ 1.5 nm^−1^), which appear to describe high-quality simulation data better than the original theory and permit determining ${\bar{\kappa }}_{m}$ via fitting—see figure 5. This is useful not so much for its application in simulations, where the second moment of the stress profile can be measured directly, but rather for future experiments, because high-q shape undulations can be accessed via x-ray scattering [35].

Finally, the importance of tilt for high-curvature structures such as the stalk [26], and the deviations observed at high q-vectors, where curvatures become comparable to tilt decay length and pivotal plane distance, suggests that higher (i.e. beyond quadratic) order corrections to continuum theory contribute noticeably to the energetics of fission and fusion. The existing continuum-elastic framework [29, 34] can be extended in this way; the challenge will be to do this consistently.

### Concluding remarks.

A random number between 0 and 500 k_B_T is unlikely to be close to k_B_T. Unless cells choose their lipid composition accordingly, we should expect the energy barrier towards fusion to be dominated by topological Gaussian curvature energy. Hence, the fundamental energetics of innumerable membrane remodeling events in cells must be characterized by at least one of the following two: (i) an ingenious protein machinery that evolved to wrestle with the demand for close-to-instantaneous supply of significant topological curvature energy, and (ii) an ingenious tuning of lipid composition that lowers this topological barrier $|4\pi \bar{\kappa }|$ and makes the proteins’ tasks less formidable. Either of these solutions would be remarkable; in fact, it seems almost inconceivable that evolution only stumbled across the first one. However, since $\bar{\kappa }$ is so difficult to measure, we know close to nothing about the second option and therefore have largely focused on the first. Considering our continual progress in experimentation technology, as well as recent theoretical and computational advances, the time has come to also explore the second option more seriously.

Acknowledgments

The author would like to thank Mingyang Hu, Mert Terzi, and John Nagle for many useful discussions. Financial support by the NSF (CHE #1464926) is also gratefully acknowledged.

## Measuring the membrane spontaneous curvature

Rumiana Dimova

Department of Theory and Bio-Systems, Max Planck Institute of Colloids and Interfaces, Science Park Golm, 14424 Potsdam, Germany

### Status.

**Figure 6.**
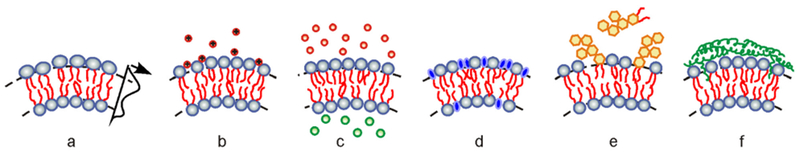
Sources of nonzero spontaneous curvature include: (a) differences in the effective head-group size (and respective molecular area) of the lipids in the bilayer, e.g. as a result of differences in hydration, pH or molecular structure of the constituting species; (b) asymmetric ion distribution leading either to condensing or expanding the lipids in one of the bilayer leaflets; (c) asymmetric distribution of nonadsorbing particles or (bio)molecules of different sizes; (d) amphiphilic molecules or lipid species asymmetrically distributed in the membrane; (e) partially water-soluble molecules (such as glycolipids or peripheral proteins) asymmetrically inserting in or desorbing from the membrane; (f) asymmetrically inserted/anchored proteins with specific geometry.

**Figure 7.**
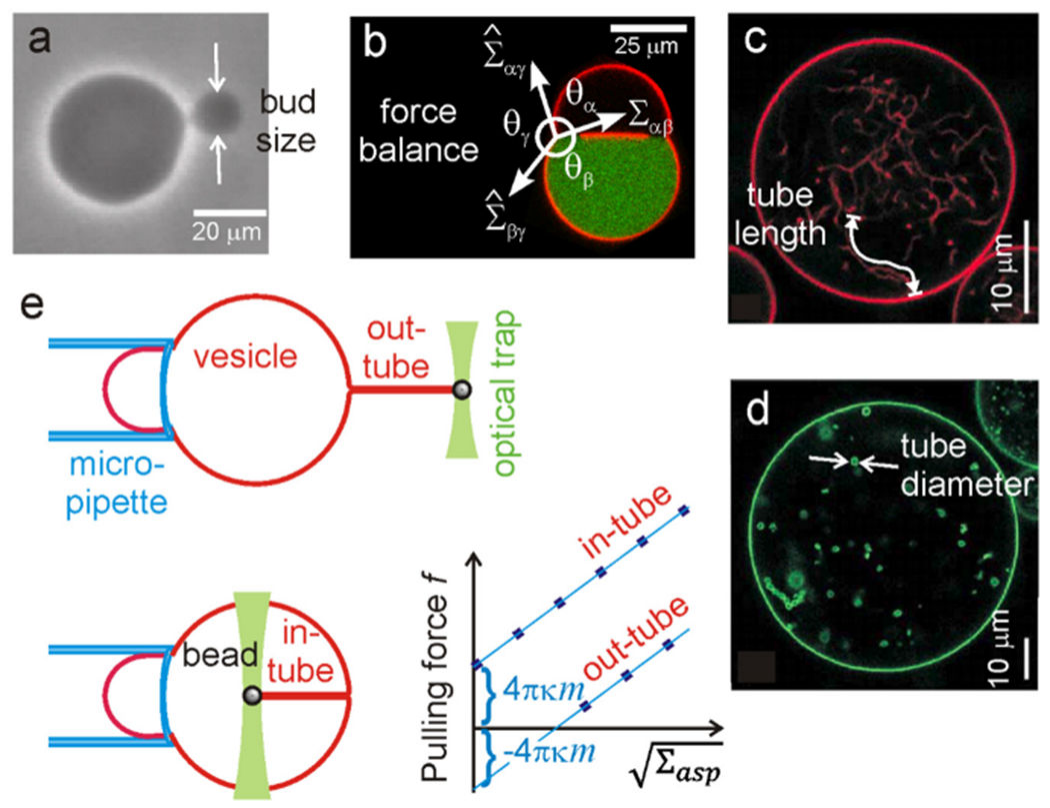
Schematic presentation of experimental approaches for measuring the membrane spontaneous curvature: (a) the spontaneous curvature of a budded vesicle can be directly assessed from its geometry following m = (M_bud_ + M_ves_) /2, where M_bud_ and M_ves_ are the mean curvatures of the bud and the mother vesicle, respectively. (b) Force balance at the three-phase contact line in vesicles encapsulating two aqueous phases, *α* and *β*, yields a direct dependence of the spontaneous curvature $m=-\sqrt{\frac{\sum_{\alpha \beta }}{2\kappa }\frac{\sin\theta_{\beta }}{\sin\theta_{\gamma }}}$ on the geometric angles, the interfacial tension Σ_*αβ*_ and the membrane tensions ${\hat{\Sigma }}_{\alpha \gamma }$ and ${\hat{\Sigma }}_{\beta \gamma }$ [37]. (c) The area stored in internal tubes and their length as measured from 3D scans can be used to assess the tube diameter and thus the membrane spontaneous curvature. (d) Tube diameters can be directly measured when they are above the optical resolution. In both approaches (c) and (d), the spontaneous curvature is |m| = 1 /(R_sph_) for necklace tubes where R_sph_ is the radius of the composing spheres or |m| = 1/ (2R_cyl_) for cylindrical tubes with radius R_cyl_; the sign of m is negative for inward tubes and positive for outward ones. (e) Pulling outward and inward tubes of a vesicle held by a micropipette at tension Σ_asp_ and measuring the pulling force f applied by an optical tweezer yields the spontaneous curvature $m=\mp \frac{f}{4\pi \kappa }$ from the *y*-axis intercept of force data as sketched in the inset for membranes with positive spontaneous curvature (the membrane bending rigidity *κ* is assessed from the slopes of the data dependence) [38, 39].

All biomembranes exhibit or are exposed to asymmetry. This asymmetry can originate from the composition of the leaflets [36] but also from their immediate environment in terms of solution composition. Any type of asymmetry across a membrane will influence its spontaneous curvature and thus the preferred membrane shape. It is then obvious that shapes of membrane organelles and cellular protrusions can be directly influenced by spontaneous curvature. The most ‘popular’ and sought for sources of asymmetry and thus membrane remodeling factors are proteins which bind to the membrane and reshape it as discussed in the section of Bassereau (section 1) and the section of Jin and Baumgart (section 2) in this Roadmap. However, any type of substance, as long as it is asymmetrically distributed across a membrane, will affect the membrane spontaneous curvature. Such substances include ions, particles and water soluble (macro)molecules, even those that are conventionally considered as inert to the membrane, e.g. polyethylene glycol. Only perfectly symmetric membrane leaflets and transmembrane solution compositions and asymmetric systems with perfectly balanced intermolecular interactions in both leaflets and solutions can result in a membrane of zero spontaneous curvature. This ideal case is practically irrelevant because every biological membrane experiences asymmetry of various origins as exemplified in figure 6. Any deviations from this ideal case, including for example local compositional changes (clusters and domains) in one of the leaflets, will result in generation of nonzero local spontaneous curvature. This, in turn, can exhibit itself in spontaneous budding or tubulation of vesicles [37–39], see also figures 7(a), (c) and (d).

Measurements of the membrane spontaneous curvature are relevant as they give an idea of the preferred shape the membrane will take in a relaxed state or in the absence of applied tension. Presumably, the most practical system to employ for the purpose of assessing this material property are giant unilamellar vesicles (GUVs) [40]. One of the first attempts to measure the membrane spontaneous curvature, m, addressed the effect of sugar asymmetry across the membrane [41], where m was assessed from the fluctuation spectra of GUVs yielding m^−1^ ~ 10 ÷ 100 *μ*m. A later work examining the effect of asymmetrically anchored biopolymers estimated the spontaneous curvature from the size of generated buds [42] m^−1^ ~ 1 ÷ 10 μm, see also figure 7(a); note that this approach can be applied provided the compositions of the bud and the mother vesicle membranes are identical. The magnitude of the spontaneous curvature can vary from several inverted microns (as in the above-mentioned cases) to few tens of inverted nanometers, m^−1^ ~ 20 ÷ 100 nm, as is the case of BAR domain proteins [43]. Intermediate values are found for membranes asymmetrically exposed to divalent ions [44, 45] or polymers such as polyethylene glycol, m^−1^ ~ 0.1 ÷ 0.3 *μ*m [37]. The effect of the latter has been assessed in two ways: (i) from estimating the diameters of necklace-like or cylindrical tubes in GUVs (either from direct measurements on relatively thick tubes or deducing the tube diameter from the area stored in tubes and their total length as tediously tracked from 3D confocal scans), see figures 7(c) and (d), and (ii) from force balance at the three-phase contact line in vesicles exhibiting aqueous phase separation in their interior, figure 7(b). Measurements on systems with high spontaneous curvature typically rely on pulling lipid nanotubes out of GUVs [46]. One approach is illustrated in figure 7(e) [39].

Note that the membrane spontaneous curvature represents a material property of the membrane and should not be confused with reported values of ‘molecular curvature’, which is typically related to the molecule geometry and is strongly influenced by its environment, see the next section.

### Current and future challenges.

Once the spontaneous curvature is measured, a challenging task is to correlate, in a quantitative manner, the membrane interactions and behaviour at the nanometer scale with the morphological response at the micrometer scale in vesicles and in cells. For molecules inserted in the membrane, it is attractive to visualize the origin of the spontaneous curvature as some sort of molecular geometry: cone-shaped molecules inserting in the outer leaflet will generate positive spontaneous curvature, and inverted cones—negative. Spontaneous curvature of protein-doped membranes as assessed from tube pulling experiments are also interpreted in terms of some ‘effective spontaneous curvature of the protein’ after taking into account its surface density, see e.g. [46]. However, the studied molecules themselves would not form a surface with specific curvature and the obtained parameter represents rather a local curvature generated by the protein; it is a material property that is not universal but depends on the molecule environment. Thus, reported values should not be generalized as they are specific to the explored system and membrane composition.

Another challenging task is to convey curvature-generation information obtained at the level of simple model bilayers to more complex cellular membranes. The hope is that the effects measured on ‘simple’ membranes may be translated to highly complex bio-membranes because of e.g. equivalent elevated local concentrations of the curvature-triggering factor such as proteins or ions.

The spontaneous curvature of membranes are predominantly examined on GUVs as model systems. Their membranes are typically multicomponent in studies mimicking the cellular conditions. However, the methods for preparing giant vesicles do not necessarily result in membranes in which the multicomponent lipid mixture is exactly reproduced in the resulting vesicle (for example, the membrane composition can be altered when budding of a phase-separated region occurs prior to vesicle observation, or cholesterol can be depleted from the lipid mixture when phase-transfer methods are employed for the vesicle preparation). The lipid species may also end up asymmetrically distributed in the two leaflets as discussed below.

An initial step of some vesicle preparation procedures is the deposition of the lipids on a substrate (e.g. in vesicle electroformation) to which different lipids may have different affinity. Their distribution across the bilayer may also be influenced by an externally applied potential and the resting surface potential. Both effects will result into asymmetric distribution of the lipids in the two membrane leaflets [47]. While the fast flip-flop time of cholesterol ensures its redistribution in the bilayer, the slow transbilayer mobility of lipids on typical experimental time scales may prevent their symmetric redistribution. Presumably, such an asymmetry could be one of the plausible sources for conflicting results on the calcium-induced curvature generation in GUVs composed of neutral and charged lipids. One study has reported that binding of calcium ions to these membranes generates positive spontaneous curvature [44], whereas other measurements at the same conditions displayed negative spontaneous curvature [45]. The vesicles in the first study were used fresh (plausibly asymmetric membrane), while in the second, they were left to equilibrate (presumably symmetric).

### Advances in science and technology to meet challenges.

For the characterization of the molecular origin of spontaneous curvature in specific systems, molecular dynamics simulations can be employed as recently demonstrated for the interaction of polyethylene glycol with membranes of different composition and asymmetric polymer concentration across the membrane [37]. The advances in developing better models, especially when it comes to assessing the effect of partially water-soluble molecules will certainly improve our understanding of the molecular origin of the spontaneous curvature.

To resolve the molecular basis of curvature generation of proteins, biochemical approaches altering the protein structure could also be applied to reveal the contribution of e.g. transmembrane helices, protein oligomerization and scaffolding [43].

As demonstrated in figure 7, the spontaneous curvature can often be measured directly from the geometry of membranous structures such as buds and nanotubes. It is then conceivable that higher microscopy resolution would make these measurements straightforward for highly curved structures. Indeed, the advancement of super-resolution microscopy techniques such as stimulated emission depletion (STED) microscopy could make such measurements feasible. In addition, they will probably become possible *in vivo.*

### Concluding remarks.

The shape of organelles is strongly influenced by the mechanical properties of membranes. Even membraneless organelles, thought to form via thermodynamic cues, occasionally come in contact with the abundant membranous organelles in cells. Being wetted by a different aqueous phase, the membrane will experience strong asymmetry and the generated spontaneous curvature and associated tension (~ 2*κ*m^2^) will most likely result in reshaping the membrane. This once again emphasizes the importance of understanding the origin and assessing the magnitude of spontaneous curvature.

Acknowledgments

The author would like to thank Reinhard Lipowsky for many useful and inspiring discussions. This work is part of the MaxSynBio consortium, which is jointly funded by the Federal Ministry of Education and Research of Germany and the Max Planck Society.

## Mechano-chemistry and catalysis of membrane fission: lessons from dynamins

Vadim A Frolov^1,2^ and Pavel V Bashkirov^3,4^

^1^ Biofisika Institute (CSIC, UPV/EHU) and Department of Biochemistry and Molecular Biology, University of the Basque Country, Leioa 48940, Spain

^2^ IKERBASQUE, Basque Foundation for Science, Bilbao 48013, Spain

^3^ Federal Research and Clinical Centre of Physical-Chemical Medicine, Moscow 119435, Russia

^4^ A.N. Frumkin Institute of Physical Chemistry and Electrochemistry, Russian Academy of Sciences, Moscow 119071, Russia

### Status.

**Figure 8.**
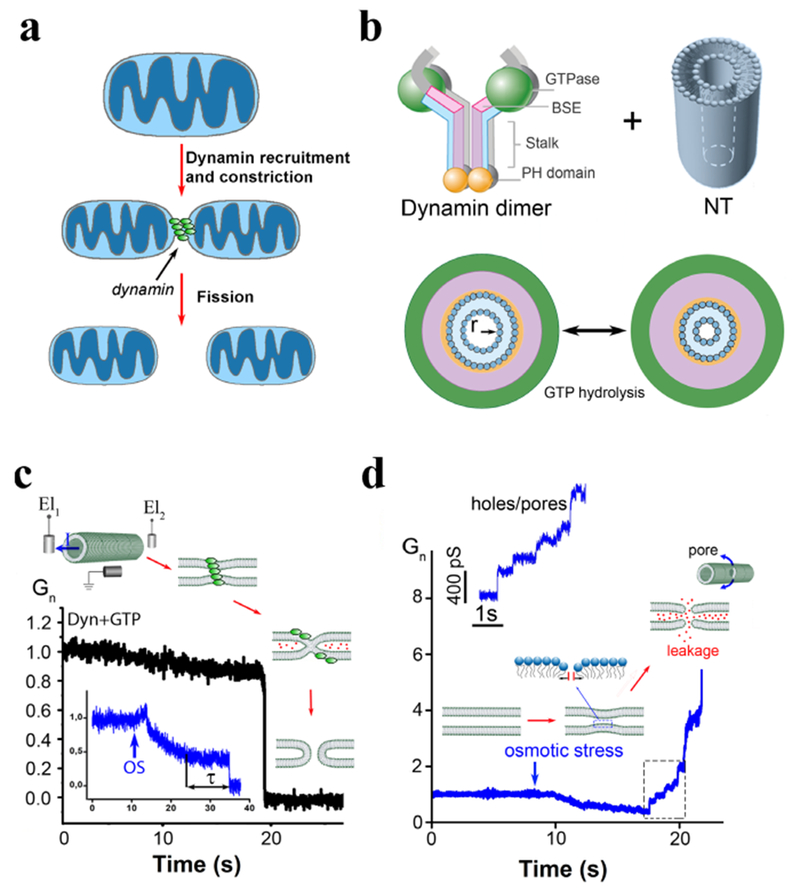
Phenomenology and pathways of dynamin-driven membrane fission. (a) Dynamins in mitochondrial division. (b) Dyn1 dimer has a characteristic mushroom architecture with GTPase domains connected to membrane-interacting PH domains via a rigid stalk responsible for Dyn1 self-assembly into a helix on the surface of a lipid nanotube (NT). The mechano-chemical energy transduction is mediated by the BSE arms: their rotation causes constriction of the helix and also affect membrane interaction of the PH domains [48]. (c) Non-leaky membrane fission by Dyn1 (black) and osmotic pressure (blue) assessed by measurements of the ionic conductance (G_n_, normalized to the value before Dyn1/pressure application) of the NT lumen measured by 3-electrode scheme (see [52] for details). The cartoon illustrates the hemi-fission pathway of membrane remodeling. (d) Pore formation in the NT wall caused by the NT constriction by osmotic pressure. The cartoon outlines a possible packing defect leading to nucleation of a pore in the NT wall.

Dynamins are mechano-enzymes converting chemical energy into membrane deformations. Dynamin superfamily has been closely associated to emergence and development of intracellular organelles and membrane transport systems [51]. The superfamily has been linked to a number of severe human pathologies, explaining the sustained interest of the biomedical research community [52]. The oldest dynamin, Drp1, is widely considered as a major component of mitochondrial division, probably, since the endosymbiosis. The toughness of the mitochondrial double membrane envelope is reflected in the Drp1 approach: it oligomerizes on the neck of a dividing mitochondrion to relay the energy obtained from cooperative GTP hydrolysis to constriction and fission of the neck (figure 8(a)). Similar mechano-chemical principles characterize the whole superfamily [51]. Historically, they are best understood for dynamin 1 (Dyn1), the founding member of the superfamily involved in membrane fission during synaptic vesicles recycling [53]. As most of dynamins, Dyn1 retains its functionality in minimal *in vitro* systems [51, 53]. Reconstitution of membrane fission with purified Dyn1 and lipid membrane nanotubes revealed authentic ‘molecular machine’ that keeps fascinating physicists: Dyn1 self-assembles into a helix which internal radius is controlled by GTP binding and hydrolysis, thus defining mechano-chemical action in fission ([53], figure 8(b)). The elementary helical unit, Dyn1 dimer, has major molecular motor attributes: GTPase head and the relay mechanism transmitting the nanoscopic conformational rearrangements of the GTPase domains during the hydrolysis cycle into macroscopic transformations of the Dyn1 helix ([53, 54], figure 8(b)). Dyn1 also has molecular switch attributes: its membrane interactions depend on the nucleotide binding state [48]. Such regulation might be a defining feature of fission proteins [55]. In Dyn1, it is mediated by the Plekstrin homology domain (PHD). PHD regulates on-membrane self-assembly of Dyn1 and has also been implicated in catalysis of membrane fission [50, 56]. PHD is lacking in earlier dynamins, illustrating functional evolution of dynamin superfamily [54]. On the other hand, during the last few decades, it has been revealed that cells often rely on much simpler fission mechanisms, based upon curved protein molecules imposing local membrane geometry and even upon entropic force [57]. In principle, such mechanisms should be more robust than dynamins, thus questioning the widespread involvement of dynamin superfamily in intracellular membrane remodeling [53]. In part, the centric role of dynamins is related to their deep integration into intracellular networking. However, the mechano-chemical mechanism of membrane remodeling employed by them might have its own reasons to stand out.

### Current and future challenges.

**Figure 9.**
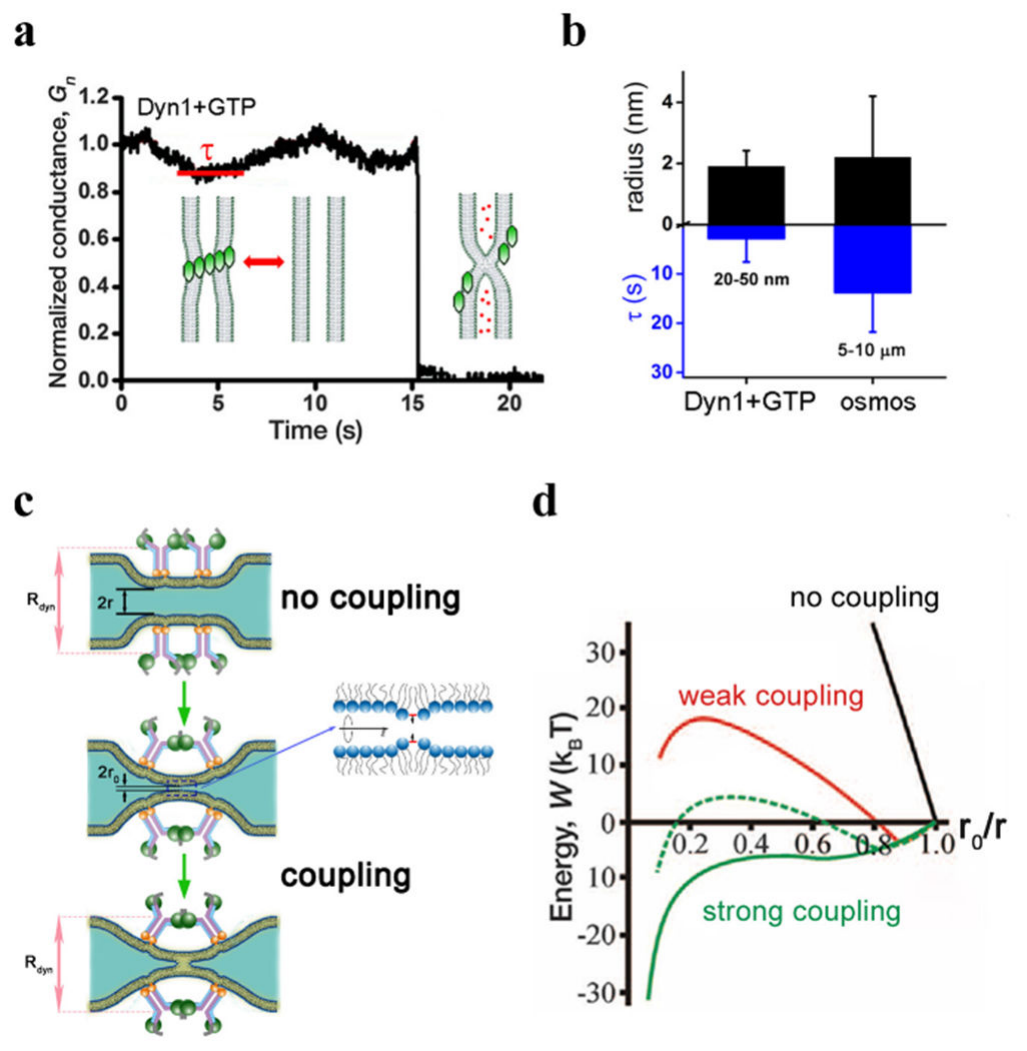
Mechano-chemistry and catalysis for non-leaky membrane fission. (a) Quasi-periodic NT constriction seen on ultra-short (80–200 nm in length) NT; G_n_ and *τ* in the constricted state (marked by the red line) characterize the size and efficiency of the Dyn1 minimal fission machinery [50]. (b) Luminal radius (black) and total time in the constricted state before fission (Σ*τ*_i_) for Dyn1- and osmotic pressure-mediated fission. The estimated length of the fission site (the constricted part of the NT) is indicated below the bars. (c) Catalysis of the hemifission transition by enabling ‘free’ tilting of Dyn1 scaffolding (at fixed *R_dyn_*) leading to optimization of the NT geometry in the constricted part that adapt less stressed saddle-like shape. The geometry change promotes interaction between packing defects in the inner monolayer of the NT resulting in its self-merger, the hemi-fission (see [51] for details). (d) Changes in the elastic energy of the NT along the constriction pathway parameterized by the NT radius in the narrowest point (r_0_). Disappearance of the energy barrier for constriction at high coupling regime underlies the catalytic effect of the coupling between the dynamin’s and membrane geometry.

To understand specifics of the mechano-chemical mechanism, a comparative analysis would be desirable. The major challenge here is to resolve the actual pathway of membrane remodeling during fission mediated by different agents. Measurements of the luminal conductance of a lipid nanotube during Dyn1-driven constriction and fission highlight the challenge: slow conductance decrease reporting the constriction contrasts with an acute, sub-millisecond conductance drop (figure 8(c)) during which the whole topological transformation completes. Dissecting this fast nanoconfined process into distinct stages is hard if possible at all. Yet, analyses of the available readouts, such as the luminal conductance, provide a viable alternative. In different experimental systems, including *in vivo* conductance measurements, the conductance drop during fission has small but finite value pointing out to a curvature instability [58]. Importantly, Dyn1 and osmotic stress cause the instability at similar luminal radii, comparable with the lipid monolayer thickness (*h* = 2 nm, [49], see also figure 9(b)). This similarity implies a *universal* curvature threshold for fission [58]. Yet we revealed an important distinction: constriction by osmotic pressure, but never by Dyn1, caused membrane poration and instability (figure 8(d)). Contents leakage and material exchange between inner and outer membrane leaflets allowed by such pores could have dire physiological consequences (such as triggering of apoptosis) in dynamin-driven fission. Hence, the complex design of dynamin fission machinery might be directed to quenching of structural instabilities associated with constriction, such as nucleation of a pore.

Pore formation was extensively studied in low-curved lipid bilayers (e.g. [59]). A defect-driven process was revealed, where application of lateral tension (*σ*) facilitated nucleation of packing defects in lipid lamella (figure 8(d)) that act as primers for the pore formation [59]. Similar defect-driven mechanism, but with the packing defects caused by bending stresses, was implicated in membrane fission [50]. In a curved membrane nanotube, the defect nucleation is likelier in the inner leaflet subjected to higher curvature stress. The defects can interact leading to self-merger of the inner layer, producing so-called hemi-fission state (figure 8(c)). Topologically, the hemi-fission defines the only non-leakage pathways of fission [49, 53]. The bending energy (per molecule) associated with hemi-fission instability in the nanotube system can be estimated in the linear elastic approximation as *ka/h*^2^, where *a* is the molecular area in the membrane plane and *k* is the mean curvature bending modulus of the nanotube membrane [58]. This energy defines the major barrier for membrane remodeling in fission. Crucially, comparable energies (estimated as *σa*) can be obtained in planar membranes under elevated lateral tension (*σ*) causing poration [58]. This similarity might account for the coupling between hemi-fission and poration in osmotic pressure-driven constriction, though quantitative characterization of both processes is pending. Of note, while osmotic pressure and Dyn1 impose the same net membrane constriction, the differences in the local deformation fields can account for the leakage occurrence. Hence, the challenge remains to resolve membrane deformations at molecular scale.

### Advances in science and technology to meet challenges.

In an attempt to better quantify nanoscale membrane deformations, Dyn1 fission was reconstituted on extremely short lipid nanotubes (80–200 nm), comparable in length with the neck of an endocytic vesicle. In this system, conductance measurements revealed quasi-periodic nanotube constriction coupled to GTP hydrolysis (figure 9(a)). This intermittent constriction pattern indicates that Dyn1 might avoid leakage by minimizing the time of curvature stress application. Indeed, quantitative comparison of Dyn1- and osmotic-driven fission revealed crucial differences in the length and time scales of the process (figure 9(b)). Cyclic membrane constriction by Dyn1 requires constant energy input, explaining the need for GTP hydrolysis in leakage-free fission. Further analysis revealed that in addition to the intermittent constriction Dyn1 also utilizes a catalytic strategy to facilitate fission while in the constricted state [51, 56]. It is plausible that this mixture of mechano-chemical and catalytic activities constitutes the mechanistic basis for leakage-free fast fission mediated by Dyn1.

The catalytic activity is mediated by PHD. PHD are large entities [53] and with their position fixed by Dyn1 helix they can severely restrict membrane geometry [50, 56]. Nevertheless, theoretical analysis predicted that allowing free tilting of PHD could dramatically facilitate the hemi-fission transition at a fixed radius of Dyn1 helix (*R*_*dyn*_, figures 9(c) and (d)). The strength of this effect depends on the PHD-membrane interactions. In the strong coupling regime when PHD explicitly imposes the geometry of lipid monolayer beneath it, the energy barrier for the hemi-fission transition disappears, revealing a catalytic soft mode [50]. Alternatively, PHD tilting can help converting radial constriction into an axial force needed to rupture the hemi-fission intermediate [48, 60]. Substantial PHD tilting can only occur at the ends of Dyn1 helix so that the tilting would be the most effective in a 2-rung Dyn1 scaffold (figure 9(c)). Crucially, this short scaffold was experimentally identified as the minimal Dyn1 fission machinery [50]. Its geometry matches that of the saddle-like hemi-fission intermediate ([50, 58], figure 9(c)) implying that Dyn1, as catalysts in general, specifically recognizes the structure of the major transition state of the fission reaction.

The minimal Dyn1 machinery still contains tens of the protein molecules acting upon hundreds of lipids (figure 9(b)). Studying such mesoscopic systems, too large for single molecule approaches but too small for ensemble analyses, remains a major technological challenge. An emergent approach to such systems is high-speed atomic force microscopy (AFM) capable of resolving conformational dynamics at single molecule level [61]. Yet, creation of adequate lipid template enabling high resolution AFM imaging of membrane fission would be required to advance the technique. Alternatively, molecular re-engineering of proteins, swapping functional blocks and introduction of artificial mechanical parts (such as cross-linkers, [48]), should facilitate functional analysis of the membrane fission machineries.

### Concluding remarks.

Typical schematic of dynamin fission machinery depicts a hose-clamp mechanism. Gradual tightening of the clamp shall eventually produces membrane disconnection making the mechanics of the clamp tightening the major puzzle in dynamin-driven fission. Yet, as we remind here, increasing curvature stress can produce membrane disconnection by various means, including membrane rupture. It seems that the functional design of the dynamin machinery takes into account this undesirable instability. Instead of a stress ramp, dynamins apply intermittent constriction somewhat resembling cyclic fatigue approach. Equipped with a catalytic core greatly facilitating hemi-fission transition upon constriction, modern dynamins can effectively produce fission without risking leakage, thus imposing the spatial and temporal controls required by physiology onto stochastic, defect-driven membrane remodeling process.

Acknowledgments

The work was partially supported by Spanish Ministry of Economy and Competitiveness (Grant BFU2015-70552-P) and the Russian Science Foundation (Grant No. 15-14-00060).

## Steric and energetic challenges in SNARE-mediated fusion of membranes

Helmut Grubmüller^1^, Reinhard Jahn^2^ and Jelger Risselada^3,4,5^

^1^ Department of Theoretical and Computational Biophysics, Max Planck Institute for Biophysical Chemistry, Göttingen, Germany

^2^ Department of Neurobiology, Max Planck Institute for Biophysical Chemistry, Göttingen, Germany

^3^ Department of Theoretical Physics, Georg-August University, Göttingen, Germany

^4^ Leiden University, Leiden Institute of Chemistry, The NetherLands

^5^ Leibniz Instite of Surface Modification, Chemical Department, Leipzig, Germany

### Status.

Eukaryotic cells contain membrane-enclosed organelles that communicate with each other by the exchange of trafficking vesicles. Each trafficking step consists of the generation of a transport vesicle from a precursor compartment involving budding and fission, the transport of the vesicle towards its destination membrane, and finally the docking and fusion of the vesicle at the target membrane.

Membrane fusion of trafficking vesicles is mediated by SNARE proteins, which comprise a family of small membrane proteins. They contain evolutionarily conserved sequence motifs of 70–80 residues, termed SNARE motifs that form four distinct subfamilies. Usually, the SNARE motif is connected by a short linker to a C-terminal transmembrane domain. The N-terminus often contains an additional domain that serves both as recruiting device for regulatory proteins and/or as auto-regulatory domain by binding to the SNARE motif. Some SNAREs deviate from this general structure, e.g. by lacking a transmembrane- or an N-terminal domain, or by containing two linked SNARE motifs.

Assembly of four SNARE motifs, one from each subfamily, into SNARE complexes contributes the prime energy source for membrane fusion. Complementary sets of SNAREs assemble in ‘trans’ between the membranes, with assembly initiated in the N-terminal region of the SNARE motif and then progressing towards the C-terminal membrane anchors, thus pulling the bilayers towards each other. Although determination of the free energy of assembly is hampered by a pronounced hysteresis, estimates, based both on ensemble and on single molecule force experiments, range from 13 to 27 k_B_T.

### Current and future challenges.

Despite high-resolution structures of SNAREs, SNARE complexes, and SNARE complexes associated with regulatory proteins, the molecular pathway of SNARE-mediated fusion is controversial. Open questions include the structure and composition of the fusion complex and its changes during progression along the reaction path, the geometry and structure of the fusing membranes with respect to curvature, non-bilayer intermediates, and membrane lipid arrangements, as well as the energy barriers and energy minima that need to be traversed along the reaction coordinate. In the following, we will briefly discuss some of the major challenges that need to be addressed.

### Advances in science and technology to meet challenges.

#### Steric constraints of the SNARE fusion machine.

**Figure 10.**
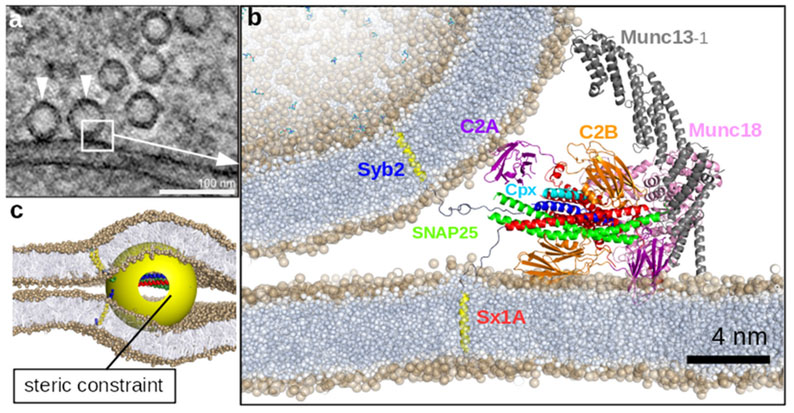
Physics of SNARE-controlled membrane fusion. (a) Electron microscopy image of lipid vesicles docked to the synaptic membrane (arrows). Reprinted from [62], Copyright 2014, with permission from Elsevier. (b) Plausible arrangement of the fusion machinery protein complex (see text) [62–64] in between the vesicle (upper left) and the synaptic membrane (bottom), indicating the steric challenge posed by the huge volume of the protein complex. (c) Coarse grained simulation snapshot: a steric constraint (yellow sphere) of similar size induces considerable curvature that promotes close approach of the apposing membranes (left), eventually inducing membrane fusion.

It is well established that assembly of SNAREs is governed by regulatory proteins belonging to the SM- and CATCHR-protein families, often in complex with additional proteins of considerable size. Examples include the HOPS complex regulating the SNAREs involved in late endosome-vacuole fusion, or the activated fusion complex involved in neuronal exocytosis. These voluminous protein complexes are thought to be bound to the SNAREs (often at the site of the SNARE motif) at least until fusion is initiated. Fusion of highly curved synaptic vesicles (with a curvature of ca. 1/20 nm^−1^, figure 10(a)) with the pre-synaptic plasma membrane already requires substantial unfolding of the SNAREs helices (figure 10(b)), with so far unknown effect on their functionality. An even larger steric challenge is posed by late endosome-vacuole fusion, where the involved curvature at the site of membrane fusion is much lower and the SNARE-bound complexes (such as the HOPS complex) are even larger. Therefore, fusion may proceed via structurally quite different pathways and intermediates, with considerable consequences for the energy landscape. Figure 10(c) illustrates this situation: Any bulky protein complex (yellow sphere) necessarily introduces high membrane curvature when the opposing leaflets are brought in close proximity (up to ca. 1 nm [69, 70]) by the SNARE transmembrane anchors to promote hemifusion. Indeed, a recent study on yeast vacuole fusion suggests that SNARE binding of non-specific artificial, soluble molecular complexes which are of similar size as the SNARE binding head region of HOPS (14 nm diameter) drives the transition of a trapped hemifusion intermediate into a fusion pore [65]. Intriguingly, these observations suggest that such a steric clash may actually be essential for the hemifusion to fusion pore transition, especially under low membrane curvature conditions. Indeed, MD simulations revealed that the curvature imposed on the hemifusion stalk reduces the free energy barrier of subsequent fusion pore formation considerably from 67 to 34 k_B_T [65].

### Membrane structure and membrane deformation immediately before transition to non-bilayer intermediates.

**Figure 11.**
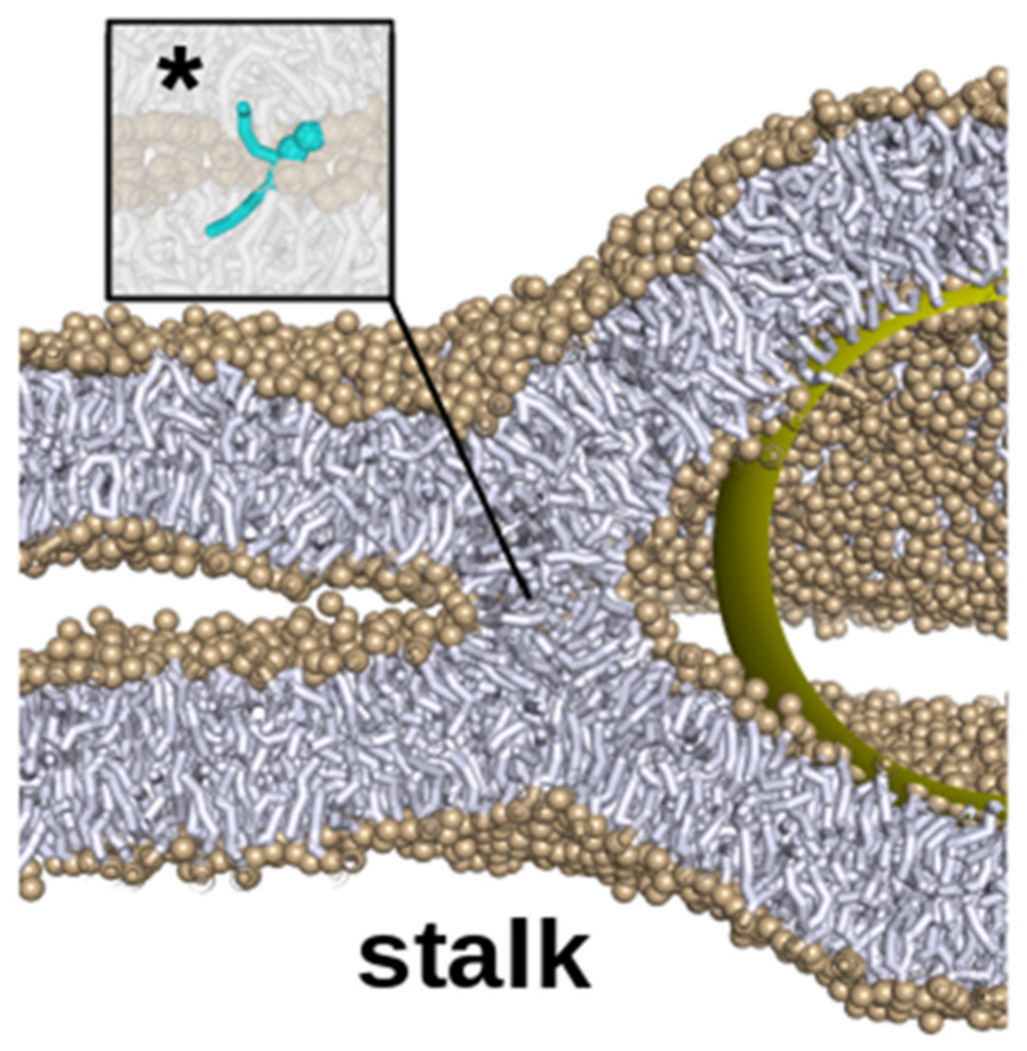
Molecular scale distances (inset) are crucial for stalk formation.

Recent studies [66, 67] revealed the presence of tightly docked vesicle-vesicle contact zones that remain metastable even after enzymatic cleavage of the SNARE complexes (which initially drove docking). This attraction has been suggested to be of osmotic origin due to sterically restricted access of ions (including their hydration shell) to the narrow gap between the apposed (net charged) lipid bilayers [68]. The fact that adhesion is not observed in the presence of a formed hemifusion intermediate or fusion pore raises the question how these docked states are mobilized for fusion. Indeed, metastable membrane adhesion in stacked bilayer arrays shows a slightly larger separation distance between 1.1 and 1.3 nm [68] than membrane fusion (rhombohedral phase formation) which requires a distance below 1 nm in order to form a crucial lipid bridge [69, 70] (see figure 11). Furthermore, adhesion very likely opposes both the formation and expansion of the fusion pore as well as the formation of hemifusion intermediates, as this would reduce the amount of favorable membrane-membrane contact area [68, 70]. On the other hand, adhesion may help to overcome steric clashes and assist both zippering and structuring of the SNARE-complex (e.g. see figure 10(b)) by providing an additional and possibly essential driving force to bring membranes in sufficiently close proximity. Which of these effects dominates under which conditions is unclear, and more quantitative studies and simulations will be required to address this issue.

### Concluding remarks.

The primary step of membrane fusion, must be preceded by an approach of the opposing membranes below a critical distance of ca. 1 nm, regardless of membrane composition [69, 70] or an imposed steric constraint. Since the barrier to hemifusion is topological, it is likely favored by a reduction in *Gaussian* curvature *elastic* energy, quite in contrast to the steric constraint on the fusion site, which is independent on *Gaussian* curvature; see the section of Deserno (section 3) of this Roadmap and [27]. Nevertheless, the details of the subsequent fusion steps at such a molecular scale distance (see figure 11) are likely not fully captured by continuum descriptions in terms of purely electrostatic effects or elasticity theory, but rather require a full molecular description for a detailed and quantitative understanding of the structural changes that govern fusion energetics and kinetics. Here, a combined approach of well controlled *in vitro* experiments, high resolution imaging, simulation studies, and free energy calculations will be essential to test the above ideas and to advance our understanding of the underlying physics of SNARE-controlled membrane fusion.

Acknowledgments

H G and R J were supported by the Deutsche Forschungsgemeinschaft, Grant SFB 803. H J R acknowledges the state of Lower Saxony for funding via the life@nano excellence initiative.

## Endocytic entry into cells—lessons from bacterial Shiga toxin

Ludger Johannes

Cellular and Chemical Biology Unit, Institut Curie, PSL Research University, U1143 INSERM, UMR3666 CNRS, 26 rue d’Ulm, 75248 Paris Cedex 05, France

### Status.

Micropinocytosis describes the uptake of small increments of extracellular fluid that occurs when endocytic carriers form at the plasma membrane of eukaryotic cells. By far the best characterized micropinocytic mechanism is the one that is driven by clathrin and its interacting partners. Yet, it is well established today that so-called clathr in independent carriers (CLICs) exist that are morphologically different from clathrin vesicles, and that continue to perform micropinocytic uptake even when the clathrin pathway is inhibited [71]. CLICs contain exogenous cargoes such as the bacterial cholera toxin, and endogenous cargoes such as the stem cell marker CD44. The mechanisms by which they are formed are just now beginning to be unraveled.

### Current and future challenges.

**Figure 12.**
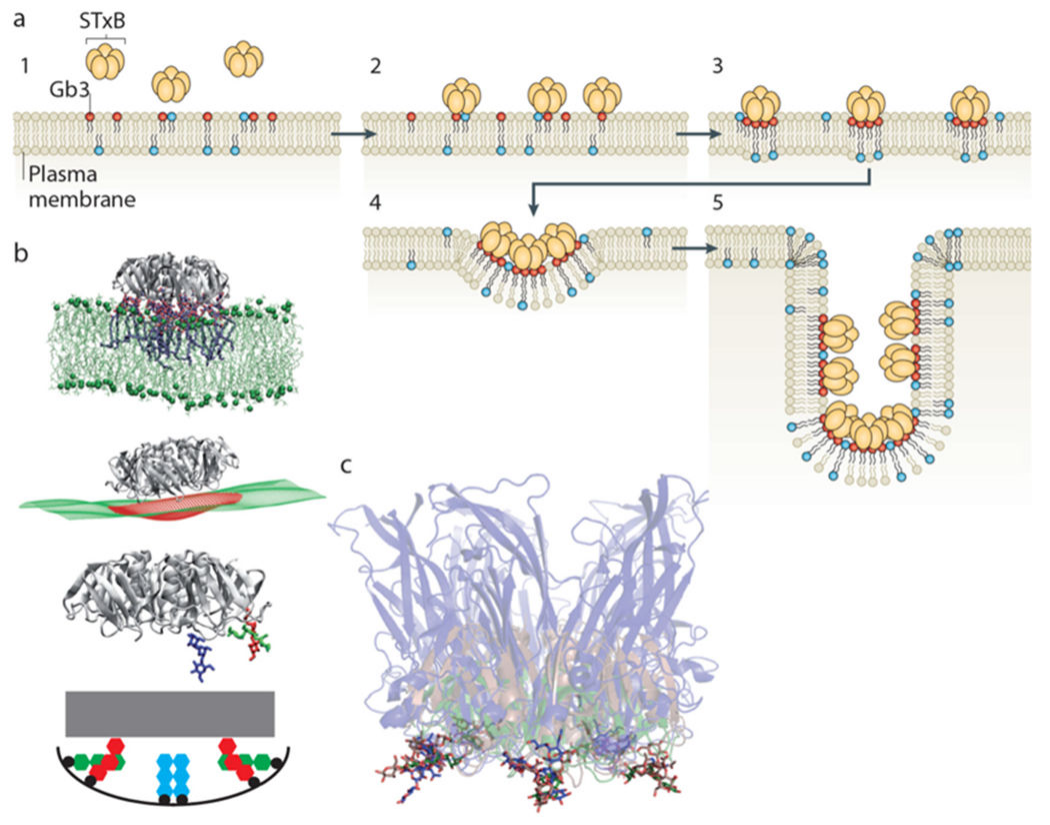
Shiga toxin-driven membrane invagination. (a) STxB (doughnut-shaped) interacts with Gb3 molecules (red head groups) in a way such as to drive the formation of tubular membrane invaginations. (b) In MD simulations, STxB also induces an increment of spontaneous curvature. See text for details. (c) The overlay of GSL receptor binding parts of Shiga toxin (green), cholera toxin (red), and SV40 (blue) present the GSLs to which they are associated with the same geometry. Reprinted from [72], Copyright 2016, with permission from Elsevier.

In 2007, it was shown that the bacterial Shiga toxin has the capacity to drive the formation of tubular membrane invaginations in interaction with its cellular receptor, the glycosphingolipid (GSL) globotriaosylceramide (Gb3 or CD77), without the need for the cytosolic clathrin machinery [72]. A model was suggested according to which the complex between the receptor-binding B-subunit of the toxin (STxB) with Gb3 lipids (note that one homopentameric STxB can bind up to 15 Gb3 lipids, i.e. three binding sites per monomer) is endowed with curvature active properties to induce an increment of spontaneous curvature (see the section of Dimova, section 4 of this Roadmap), such that deep and narrow invaginations are formed when several STxB molecules come together (figure 12(a)).

At first sight, this model appears surprising as the asymmetric load of STxB on the exoplasmic membrane leaflet would be expected to generate steric stress, to which the membrane would respond by buckling to the outside; see the section of Zeno and Stachowiak, (section 11) of this Roadmap. How does the STxB-Gb3 complex overcome this crowding effect?

### Advances in science and technology to meet challenges.

Recent molecular dynamics studies (MD) have provided clues as to how this might be achieved. In these simulations, STxB induces an increment of 0.03 nm^−1^ of spontaneous curvature [73] (figure 12(b)), as predicted by the model (figure 12(a)). The driving force appears to be the geometry of Gb3 binding sites: sites 2 and 3 of each monomer (respectively green and red in figure 12(b)) are located at the rim of STxB molecules such that the membrane needs to bend up at the edges to position the sugar parts of Gb3 molecules into these sites. Thus, despite the fact that the membrane proximal surface of STxB is as such flat, it is the geometry of the protein-lipid complex that apparently provides the bending force.

The bacterial cholera toxin and simian virus 40 (SV40) both bind to the GSL GM1 as their cellular receptor, and are both internalized by clathrin-independent endocytosis [71, 75]. Their receptor binding parts (CTxB and VP1, respectively) do not have any sequence similarity. Yet, the molecular geometry of GSL binding site 2 is conserved between these molecules (figure 12(c)). Strikingly, cholera toxin and SV40 share with Shiga toxin the capacity to induce tubular membrane invaginations [76], suggesting that this molecular architecture was selected by convergent evolution towards a same function: the generation of membrane curvature for the clathrin-independent biogenesis of tubular endocytic pits from which CLICs are formed.

Experiments on cell [77] and model membranes [73, 77] show that Shiga toxin very efficiently undergoes clustering, despite the absence of any indication for direct protein-protein interaction. For example, when for the Gb3 GSL a flexible linker is introduced between the globotriose sugar head group to which Shiga toxin binds and the ceramide part that is inserted into the membrane, the toxin molecules fail to cluster even at surface densities that are similar to the ones obtained on natural Gb3 [77]. Toxin clustering therefore appears to be membrane mediated.

**Figure 13.**
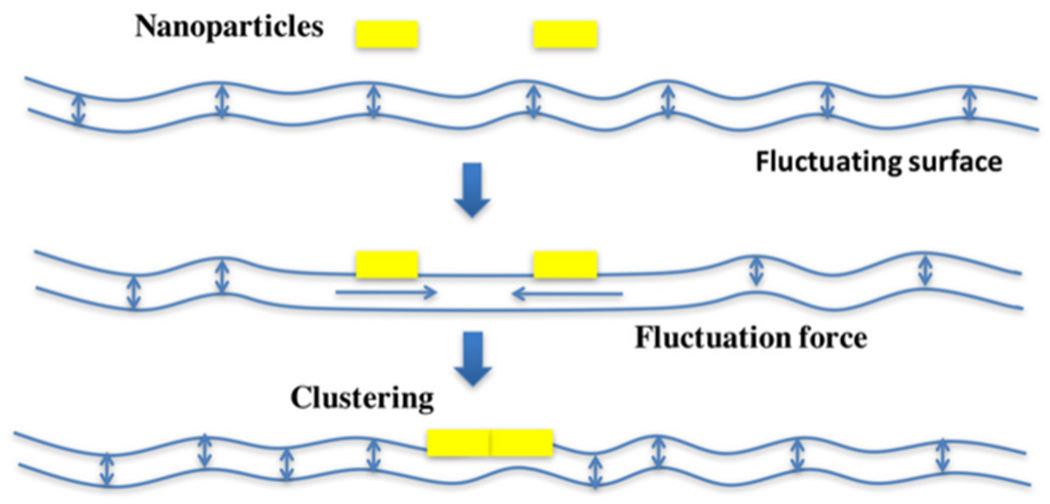
Hypothesis on fluctuation force-driven clustering. The represented nanoparticles could be Shiga toxin pentamers. Reproduced with permission from Weria Pezeshkian.

MD studies suggest that due to the presence of 15 Gb3 binding sites, Shiga toxin induces membrane nanodomains under each STxB molecule that contain up to 30 mol% of Gb3 [74]. As mentioned above, such domains would be characterized by a spontaneous curvature imprint (figure 12(b)). As such, one might expect that capillary, lipid depletion, and/or curvature forces contribute to toxin clustering, which in the cellular context is likely to be the case. However, when in model membrane experiments experimental conditions were chosen in which these driving forces were expected to be minimized, the surprising observation was made that STxB still efficiently clustered [77]. Dissipative particle dynamics coarse grain simulations (see the section of Deserno, section 3 of this Roadmap) were therefore chosen to come up with new hypothesis on the origin of this unexpected finding. In these in silico experiments, STxB was represented by rigid tightly membrane-associated nanoparticles [77], reproducing the situation predicted from the MD simulations [74]. By varying nanoparticle size, rigidity, or the flexibility of the linkers via which these nanoparticles were attached to the membrane surface it was concluded that clustering was strictly correlated with capacity of the nanoparticles to suppress membrane fluctuations (figure 13). Flexible linker experiments that were already mentioned above provided evidence that such fluctuation-induced forces might indeed drive toxin clustering on model membranes and in cells [77].

The Dutch physicist Hendrik Casimir was the first to hypothesize the existence of fluctuation-induced forces as a result of perturbation of quantal fluctuations of the radiation field by parallel uncharged metal plates in the vacuum [78]. It was then postulated that fluctuation-induced forces could also arise universally in structured fluids characterized by long range fluctuations [79]. At distances that are large when compared to the thickness of the membrane, membrane fluctuations that are well described as undulations generate a very weak attractive force between proteins that perturb them [80]. At mesoscopic distances between membrane inclusion, membrane fluctuation spectra cannot be described as simple undulations anymore. Several other degrees of freedom also need to be considered, such as peristaltic thickness fluctuations, local protrusion modes, lipid density fluctuations, and fluctuations of lipid tilt. Proteins that perturb these are expected to create a strong attractive force whose amplitude is in the range of conventional clustering forces, such as screened electrostatics or van der Waals interactions [77]. However, as opposed to the latter that are effective only at subnanometric distances, fluctuation-induced forces would be effective at distances that correspond to roughly the diameter of the inclusions that generate them, typically around 10 nm.

Why then do not all proteins in biological membranes coalesce? Two key requirements apply: (i) tight interaction with an area of membrane of more than 3 nm in diameter; (ii) conformational rigidity. Furthermore, conventional clustering forces of course continue to operate. As such, fluctuation-induced forces, which are of generic nature, may allow us to bring certain types of tightly membrane-associated proteins from nanometric to subnanometric distances at which hydrophobic or electrochemical effects that are assisted by the chemical structure of the interacting partners can then be sampled.

### Concluding remarks.

The two mechanisms that were discussed in this review—fluctuation-induced force-driven clustering and lectin-driven and GSL-dependent generation of tubular endocytic pits—likely apply beyond the world of pathogenic lectins. Indeed, recent work has shown that also the cellular galectin-3 drives the GSL-dependent biogenesis of tubular endocytic pits from which clathrin-independent endocytic carriers are generated for the cellular uptake of plasma membrane proteins such as adhesion molecules or signaling receptors [81]. The study of bacterial protein toxins such as Shiga toxin and cholera toxin has thereby enabled the discovery of molecular mechanisms that are expected to apply to a wide range of membrane biological processes. We have coined the term the GlycoLipid-Lectin (GL-Lect) hypothesis for an endocytic modality that might become an endocytic paradigm, complementary to the clathrin coat paradigm [72].

Acknowledgments

Weria Pezeshkian is acknowledged for preparing figure 13. Work in the Johannes team in the context of the theme of the current review is supported by grants from the Agence Nationale pour la Recherche (ANR-14-CE16-0004-03, ANR-14-CE14-0002-02, ANR-16-CE23-0005-02, ANR-16-CE23-0005-02), Human Frontier Science Program grant RGP0029-2014, European Research Council advanced grant (project 340485), European Union program H2020-MSCA-ITN-2014 BIOPOL, and the Swedish Research Council. The Johannes team are members of Labex CelTisPhyBio (11-LBX-0038) and Idex Paris Sciences et Lettres (ANR-10-IDEX-0001-02 PSL).

## Continuum elasticity models of membrane shaping and remodeling

Michael M Kozlov

Department of Physiology and Pharmacology, Sackler Faculty of Medicine, Tel Aviv University

### Status.

The original biological motivation for development of the continuum elasticity models of biological membranes was an attempt to reveal the physical background of the peculiar shapes adopted by red blood cells in health and in diseases [82–84]. More generally, the aim of these models was to understand in terms of mesoscopic physics the processes of generation and transformation of membrane shapes whose characteristic scales exceed substantially the membrane thickness of few nanometers. The fundamentals of the continuum description of membranes were created in the beginning of the 1970s by pioneering works of Helfrich, who used an approach of physics based on analysis of the energy of membrane bending deformation [84], and of Evans and Skalak, who employed an engineering method of direct consideration of elastic stresses developing within deformed membranes [83].

The idea of Helfrich’s theory originated from physics of liquid crystals due to an inherent physical similarity between nematic liquid crystals and lipid bilayers, which serve as structural matrices of biological membranes [84]. A bilayer exhibits, at least locally, features of a 2D fluid, whose structure is isotropic along the membrane plane but has a designated direction perpendicular to the membrane surface [84]. The seminal Helfrich Hamiltonian determines the energy of local membrane bending with respect to the flat state. The Helfrich model provided a tool for treatment of membrane shapes with curvature radii strongly exceeding membrane thickness, which covers a vast majority of biologically relevant membrane shapes. Yet, attempts have been undertaken to expand the theory to the cases of larger membrane curvatures [85]. Such extensions of the model required the introduction of additional elastic moduli inaccessible to experimental determination and had, therefore, a limited importance for applications. The Helfrich model was later developed to include the interplay between the membrane bending and area stretching-compression, which required consideration of another structural characteristic of the membrane, the neutral surface, for which the two kinds of deformations are energetically decoupled [86]. The most recent efforts were devoted to accounting for the energy of tilting the hydrophobic tails of lipid molecules with respect to the normal direction to the membrane plane [87].

The major goal of Evans and Skalak’s treatment was to provide equations for the intra-membrane balance of stresses determining the equilibrium shapes of membranes [83]. In contrast to Helfrich theory, these equations were derived for non-liquid membranes characterized, in addition to the resistance to bending, also by a resistance to deformations of 2D shear in the membrane plane. The goal of the latter feature was to account for the effects of protein networks, such as actin cortex or spectrin-actin membrane skeleton, attached to the lipid bilayers of cell plasma membranes.

The two approaches have been applied over the last four decades to analyses of membrane phenomena. While Evans–Skalak equations have been used in a relatively limited number of attempts to recover shapes of cells, Helfrich theory gave rise to a broad and fast developing field of soft-matter physics of lipid bilayers, which covered, in additions to the bilayer static and dynamic shaping [88] and the bilayer topological transformations through fusion and fission [89], also physics of lipid self-assembly into mesophases [90], the origin of inter-bilayer undulation forces [91], and bilayer–mediated forces between particles, such as proteins, inserted into the membrane matrix or attached to the membrane surface [92].

### Current and future challenges.

35 years of soft-matter physics of lipid bilayers resulted in a fairly thorough understanding of a wide range of phenomena exhibited by these systems. Yet, the original motivation of these studies, the physics behind the extremely rich world of dynamic shapes adopted by membranes of live cells, is, currently, at the early stage of its development. Exploration of the mechanisms by which proteins and intracellular force-generating machines drive the large-scale structure formation and topological transformations of membrane bound intracellular organelles is one of the major challenges for the current macroscopic physics of membranes.

**Figure 14.**
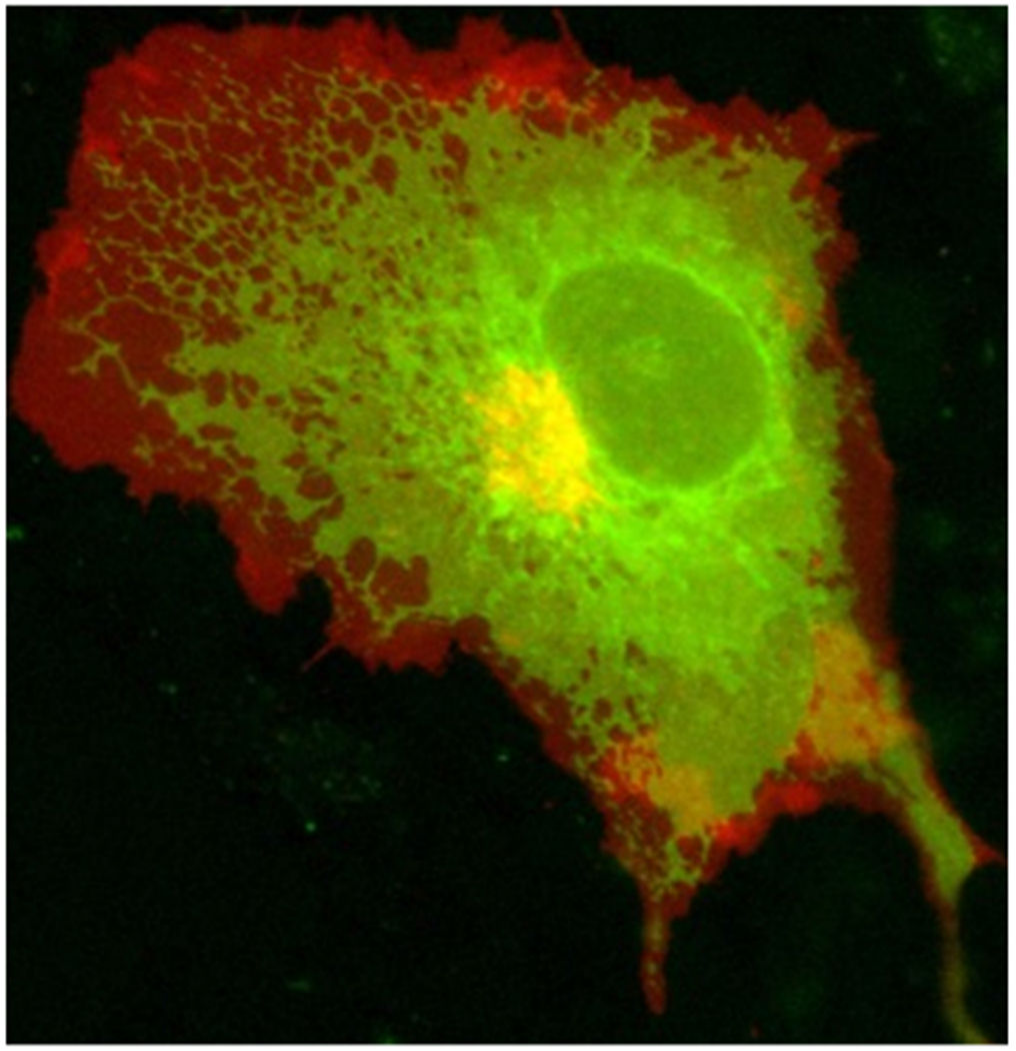
Fluorescence microscopy image of a whole cell with endoplasmic reticulum (ER) stained in green and Golgi complex (GC) stained in yellow. Reproduced with permission from K Hirschberg.

The most prominent examples of such organelles are ER and GC, the central cellular compartments representing two sequential units of the intra-cellular factory, which produces (ER) and processes (GC) proteins and lipids (figure 14). An essential part of ER and GC biological functions is to guarantee an efficient molecular communication with each other and a fast transport of their products to further destinations within the cell. This requires a unique architecture enabling, concomitantly, a large ratio between the area of the membrane covering the compartment surface and the luminal volume, a self-connectivity of the compartment lumen, a possibility of a compact packing of the compartment within the cytosol, and a high flexibility allowing for easy transformations of the compartment configurations during, for example, the cell cycle. While satisfying these general conditions, the architecture of each of these organelles needs to have individual features related to the specificity of its biochemical functions.

A common feature of ER and GC membranes enabling fulfilment of the above requirements is a substantial degree of bending of their membranes. The membrane shapes of both ER and GC include common elements—tubular, spherical, half-toroidal and saddle-like membranes of cross-sectional radii in the range of 10–25 nm, and flat membrane fragments. The modes of assembly of these elements into membrane super-structures is different for ER and GC and determines the individuality of the architecture of each of them.

**Figure 15.**
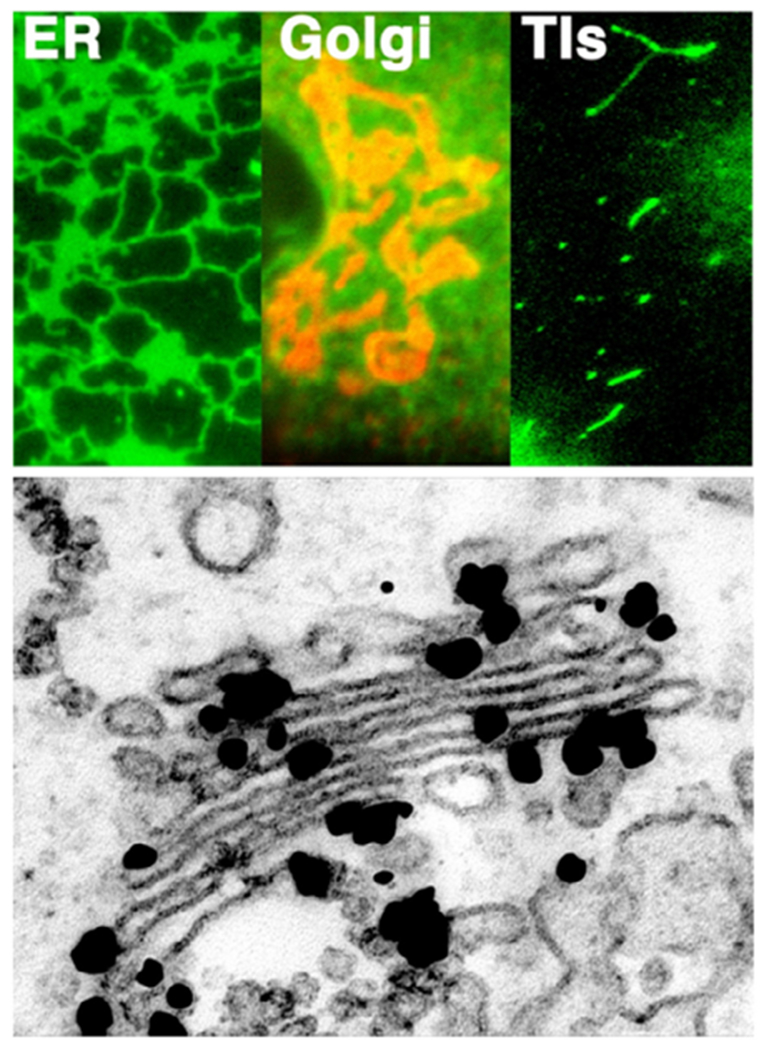
High resolution images of some intracellular organelles. Upper panel: fluorescent images of ER (left), GC (middle), transport intermediates (right). Lower panel: CryoEM image of GC stalk. Reproduced with permission from K Hirschberg.

From the physical point of view, ER of a mammalian cell is a complex membrane system consisting of a nearly spherical double membrane of the nuclear envelope (NE) and a highly-branched membrane network of the peripheral ER connected to the NE outer membrane and spanning the whole cell volume [93]. The membranes of the peripheral ER are characterized by unique shapes and complex morphologies (figure 15). The ER tubule diameters and the sheet thicknesses are close to 50 nm for mammalian and 30 nm for yeast cells, while the tubule lengths and sheet widths are in the micrometre range. The tubules and sheets are connected into elaborate networks by three-way junctions between tubules, helicoidal connections between sheets and T-like junctions between the tubule ends and sheet edges. The ER membrane network is irregular and dynamic in its structure and degree of connectivity. Three-way junctions persistently move throughout the network leading to, apparently, random collapse and de novo generation of the network elements [94]. Three-way junctions were also suggested to form dense sievelike clusters of yet unknown biological function, which could be observed only by high-resolution microscopy [95]. Finally, very recent data indicate that ER sheets may contain collections of nano-sized holes concentrated, mostly, at the sheet periphery or, sometimes, distributed all over the sheet surface. In addition to the dynamic behaviour keeping the membrane connectivity constant, the ER membranes persistently undergo events of fusion and fission changing the surface topology. The fusion reactions include such processes as merging of nascent vesicles of few nanometre size leading to de novo tubule formation, fusion between tubules resulting in 3-way junction formation, probable merger of sheets. The fission reactions encompass division of the tubules, shedding of vesicles from the free ends of tubules, probable fission of hourglass-like membrane necks forming the rims of holes within ER sheets and, possibly other types of the membrane separation. Membrane fusion and fission drive a constituent interconversion of the different types of ER morphology in the course of the cell cycle and changes of the cell metabolic state. Budding and fission of the ER membranes also mediate formation of polymorphic and strongly curved membrane elements, the transport intermediates (figure 15), which carry the newly synthesized proteins from ER to GC and other intra-cellular destinations.

The major building of GC are cisternae (figure 15)—micron wide and around 20 nm thick sheets with somewhat swollen toroidal edges [96]. The cisternae are packed in dense stacks (figure 15) which are inter-connected by long tubules forming the so-called Golgi ribbons. Also within a stack some of the cisterna are linked by tubular bridges. Each cisterna contains holes of various diameters. Some of the larger holes in different cisternae line up to form ‘wells’ within the stacks, while the others of smaller diameters (<65 nm) form fenestrations distributed all over the cisterna plane [96]. All cisternae display buds of few tens of nanometer sizes, which are, usually, associated with the cisterna rims or with the edges of the holes [96]. The transport intermediates are found in the room between ER and Golgi [96].

The physical mechanisms by which proteins generate the peculiar large scale morphologies of ER, GC and other intracellular organelles remain largely unknown.

### Concluding remarks.

In summary, while the theoretical tools of the continuum elasticity models of lipid bilayers have been developed to a high level of sophistication, they await their application to understanding the amazing world of intracellular membrane configurations and their dynamic interconversions.

Acknowledgments

M M K is supported by the Israel Science Foundation (ISF) Grant 1066/15, and holds the Joseph Klafter Chair in Biophysics.

## Curvature elasticity and multi-sphere morphologies

Reinhard Lipowsky

Department of Theory and Bio-Systems, Max Planck Institute of Colloids and Interfaces, Science Park Golm, 14424 Potsdam, Germany

### Status.

**Figure 16.**
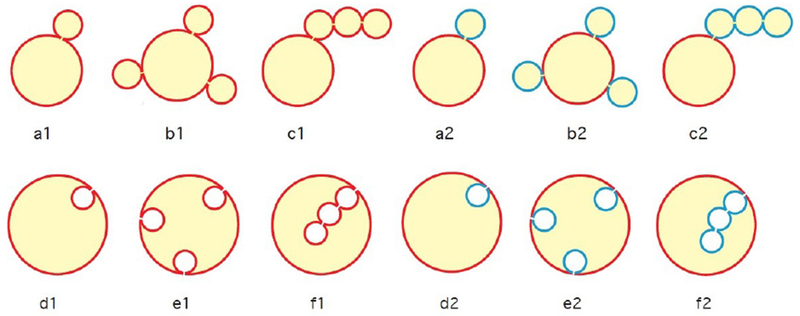
Multi-sphere shapes consisting of a spherical mother vesicle to which spherules, corresponding to small spherical buds and beads, are connected via closed membrane necks. The interior aqueous solution is yellow, the exterior one is white: (a1)–(c1) uniform membranes (red) with positive spontaneous curvature form out-buds and necklace-like tubes pointing towards the exterior solution; (d1)–(f1) uniform membranes (red) with negative spontaneous curvature form in-buds and necklace-like tubes pointing towards the interior solution; (a2)–(c2) membranes with two types of intramembrane domains (red, blue) and positive spontaneous curvature; and (d2)–(f2) multi-domain membranes (red, blue) with negative spontaneous curvature.

Lipid-protein bilayers form GUVs that attain a fascinating variety of different shapes including many distinct multi-sphere morphologies as predicted by the theory of curvature elasticity [97]. A particularly simple example is provided by budding, i.e. by the formation of a small spherical bud that is still connected to the mother vesicle via a closed membrane neck and points towards the exterior solution as in figure 16(a1) or towards the interior solution as in figure 16(d1) [98]. In these examples, the vesicle membrane was taken to be uniform in composition which implies that it has uniform curvature-elastic properties as well. Budding processes can also be induced by intramembrane domains as in figures 16(a2) and (d2). Such domains arise from lipid phase separation or from the assembly of protein coats [99], and the resulting budding processes represent essential steps for endo- and exocytosis as well as for cytokinesis during cell division.

In addition to shapes with a single bud, the vesicle can also form shapes with several buds as depicted in figures 16(b1) and (e1) for a uniform membrane and in figures 16(b2) and (e2) for a membrane with two types of domains. Furthermore, necklace-like tubes consisting of several spherical beads connected by membrane necks are also possible, see figures 16(c1), (f1) and (c2), (f2) for uniform and multi-domain membranes, respectively. In the following, buds, which are directly connected to the mother vesicle, and beads, which are connected to buds or other beads, will be collectively called spherules.

From the theoretical point of view, the multi-sphere shapes in figures 16(a1)–(f1) are primarily determined by three parameters: membrane area, vesicle volume, and preferred or spontaneous curvature m [98]. In the absence of flip-flops between the two membrane leaflets, the spontaneous curvature contains a nonlocal contribution arising from area-difference-elasticity [100]. Here, I will assume that the membrane contains (at least) one molecular component such as cholesterol that undergoes frequent flip-flops and will, thus, ignore area-difference-elasticity. In the latter case, the spherules have zero bending energy when their radius is equal to 1/ |m|, i.e. to the absolute value of the inverse spontaneous curvature. In general, we can distinguish two special classes of multi-sphere shapes: shapes with zero-energy spherules and limit shapes obtained via the closure of open necks. For positive spontaneous curvature, another type of limit shape can be formed consisting of spherules that have the same size as the mother vesicle. The latter case includes linear and branched necklace-like tubes.

The spontaneous curvature can vary over several orders of magnitude, from the inverse radius of the GUV to about 1/(10 nm) [38], which implies that the size of the zero-energy spherules can vary over the same range. All multi-sphere shapes displayed in figure 16 are stable for certain parameter regimes, which can be determined by examining the stability of the individual spheres and of the membrane necks [97].

### Current and future challenges.

Some of the shapes in figure 16 have been observed experimentally but these observations have remained fairly accidental. There are several reasons for this state-of-the-art. First of all, no serious experimental attempts have been made, so far, to control all three shape parameters—area, volume, and spontaneous curvature—simultaneously. Indeed, the standard preparation methods based on lipid film hydration and electroformation produce very polydisperse GUVs with a wide range of sizes. In addition, even though we now have a variety of methods to deduce the value of the spontaneous curvature from budded or tubulated morphologies [37, 38, 101], no reference system is currently available for which the spontaneous curvature can be varied in a systematic and controlled manner.

However, the presumably largest challenge for the preparation and observation of multi-sphere vesicles with a certain architecture is the complexity or ‘ruggedness’ of the energy landscape associated with curvature elasticity. Some insight into this landscape can be obtained by a gedankenexperiment in which we produce multi-sphere shapes with an increasing number of spherules by osmotic deflation. To be specific, let us consider a membrane with negative spontaneous curvature m < 0 that forms an initially spherical vesicle with volume $V_{0}=\frac{4\pi }{3}R_{\mathrm{ve}}^{3}$ where the overall vesicle size $R_{\mathrm{ve}}=\sqrt{A∕(4\pi )}$ is defined in terms of the membrane area A. After deflation, such a vesicle can form a variable number N of (meta)stable in-spherules with radius **R**_s_. The latter radius is somewhat variable but is always of the order of 1/ |m|. It is thus convenient to parametrize the spherule radius as **R**_s_ = *α*/ |m| with a dimensionless coefficient *α*. For *α* = 1, the spherules have zero bending energy and are always stable. Deflation of a multi-sphere shape with N zero-energy spherules increases the spherule radius **R**_s_ until we reach **R**_s_ = α_*_/|m| with 3/2 < α_*_ ⩽ 3 as follows from the combined Euler–Lagrange equations for the spherules and the mother vesicle. At this point, the in-spherules become unstable and undergo a sphere-prolate (SP) bifurcation. The precise value of α_*_ depends on the radius **R**_1_ of the mother vesicle and reaches the limiting value α_*_ = 3 for large **R**_1_.

A vesicle with N in-spherules of radius **R**_s_ = *α*/ |m| has the volume

(3) $$V(N,\alpha )=V_{0}\left({\left[1-N{\left(\frac{\alpha }{|m|R_{\mathrm{ve}}}\right)}^{2}\right]}^{3∕2}-N{\left(\frac{\alpha }{|m|R_{\mathrm{ve}}}\right)}^{3}\right).$$

**Figure 17.**
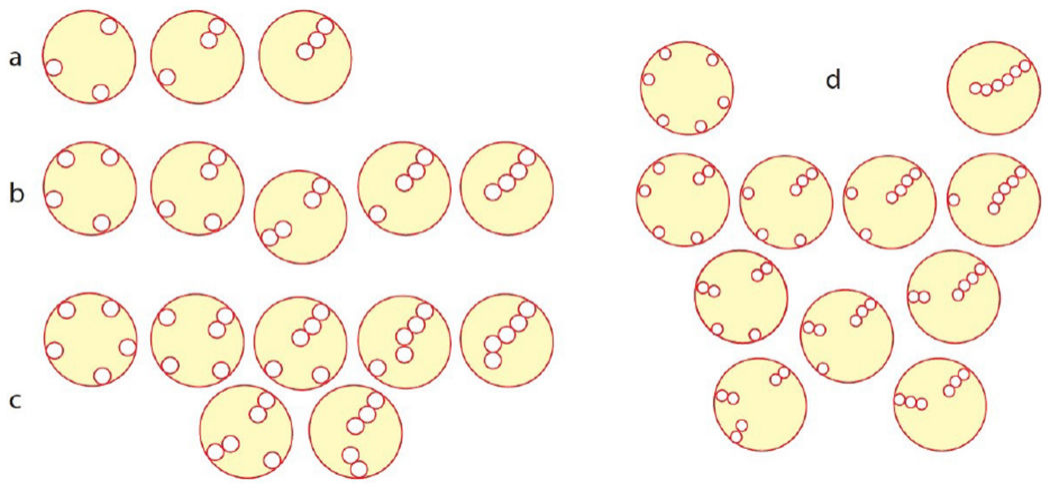
Morphological complexity emerging from multi-sphere vesicles with N spherules for negative spontaneous curvature m < 0: (a) three morphologies with N = 3; (b) five morphologies with N = 4; (c) seven morphologies with N = 5; and (d) eleven morphologies with N = 6. For given membrane area, vesicle volume, and spontaneous curvature, all morphologies with the same spherule number N have the same spherule radius R_s_ and the same curvature energy. The spherule radius R_s_ can vary between R_s_ = 1/ |m| and R_s_ = α_*_/ |m| with α_*_ < 3, see main text. For visual simplicity, all necklaces and buds have been placed into the plane of the figure, and the membrane necks connecting different spherical segments have been omitted.

It is important to note, however, that a vesicle with volume V (N, *α*) can attain, for fixed values of N > 1 and *α*, several multi-sphere morphologies as depicted in figures 17(a)–(d) for 3 ⩽ N ⩽ 6. Because the spherule radius can vary over a certain range, a vesicle with volume V(N, *α*) can also form alternative morphologies with less than N spherules and a spherule radius that exceeds *α*/ |m|.

Inspection of figure 17 reveals that we can obtain, for each N, a certain number |Ω | of distinct morphologies. This number increases from |Ω| = 3 for N = 3 to |Ω| = 11 for N = 6. In fact, for large values of N, the number |Ω| increases exponentially with $\sqrt{N}$. Furthermore, for a given membrane area, vesicle volume, and spontaneous curvature, multi-sphere morphologies with the same spherule number N have the same spherule radius R_s_ and the same curvature energy.

The morphologies depicted in figure 17 can be obtained via two basic shape transformations [37], the nucleation of a new bud via an oblate-stomatocyte (OS) bifurcation and the addition of a new bead to an existing bud or necklace via the afore-mentioned SP bifurcation. Both types of bifurcation are discontinuous and exhibit hysteresis. The OS bifurcation leads to (meta)stable spherules with radius R_s_ = *α*/ |m| and 1/(2 + *ε*) ⩽ *α* < 3 where the lower bound for *α* depends on the radius **R**_1_ of the mother vesicle via the small correction term *ε* = 1/ (|m| R_1_) ⪡ 1 as follows from the neck closure condition. Furthermore, starting from any N-spherule morphology, we can generate several distinct (N + 1)-spherule morphologies by either nucleating a new bud or extending an existing bud or necklace. In this way, we can generate the different morphologies by different sequences of OS and SP bifurcations which implies a rather rugged energy landscape. Likewise, when the closed necks of the different N-spherule morphologies are opened up by changes in vesicle volume or spontaneous curvature, we will obtain quite different vesicle shapes which again reveals that each of the |Ω| distinct morphologies with N in-spherules belongs to a different energy branch.

Repeating the osmotic deflation towards a certain volume V = V(N, *α*) several times, we will typically find different outcomes for the morphologies. When we reduce the vesicle volume to V = V(4,1), for example, we can obtain any of the multi-sphere morphologies depicted in figures 17(a) and (b) as well as intermediate morphologies with open necks. Therefore, when we perform such a deflation step many times, for the same initial volume V_0_ and the same spontaneous curvature m < 0, we expect to obtain a certain probability distribution P (S_j_|V) for the accessible multi-sphere shapes S_j_. This probability distribution reflects the underlying energy landscape and introduces a probabilistic aspect into the morphology of vesicles.

### Advances in science and technology to meet challenges, concluding remarks.

Recently, it has become possible to produce large populations of monodisperse GUVs using microfluidic double emulsions [102, 103] or pico-injection of small vesicles into emulsion droplets [104]. Furthermore, it now seems feasible to develop membrane systems for which the spontaneous curvature can be controlled in a systematic manner. Combining both developments, we should be able to produce monodisperse batches of vesicles with the same spontaneous curvature. Subsequent deflation can then produce many multi-sphere morphologies with the same volume V (N, *α*) as in (3). In this way, it should become possible to actually measure the statistics of the N-spherule morphologies and, thus, the probability distribution P (S_j_|V). Finally, it would be rather valuable to develop methods by which we can open and close the necks of multi-sphere shapes in a reversible and controlled manner. We could then develop storage and delivery systems based on these shapes.

Acknowledgments

I thank Rumiana Dimova for the opportunity to participate in this Roadmap and the MaxSynBio consortium, jointly funded by the Max Planck Gesellschaft and the Federal Ministry of Research, Germany, for a stimulating scientific environment.

## Mechanism of membrane fission: the dynamin paradigm and beyond

Thomas J Pucadyil

Indian Institute of Science Education and Research, Pune, India

### Status.

**Figure 18.**
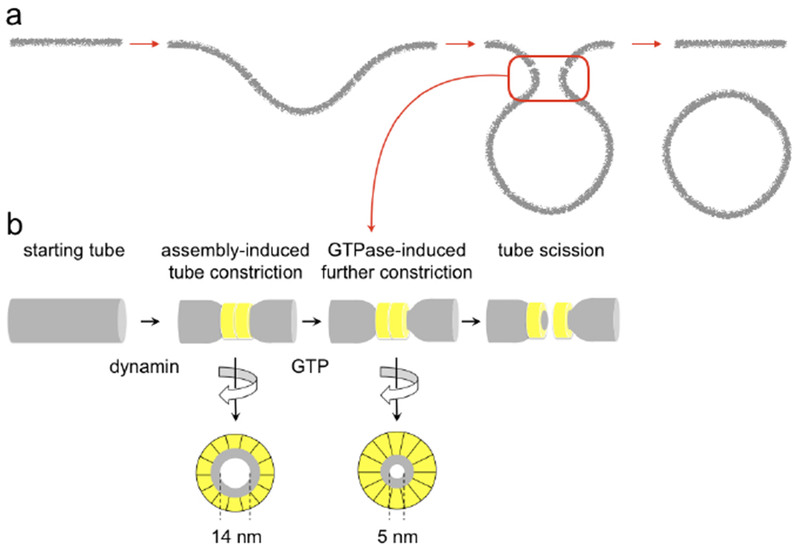
The dynamin paradigm of membrane fission. (a) The pathway to fission and release of transport vesicles follows conversion of a planar membrane into a bud-shaped structure that defines a narrow neck or a tube-like intermediate. Fission of the neck leads to the birth of a transport vesicle. (b) The pathway to dynamin-catalyzed membrane fission.

Fission or the splitting of a vesicular compartment is central to diverse cellular processes such as cytokinesis, organelle inheritance and vesicular transport. This process requires the enclosing lipid bilayer to be brought to close proximity, which, from theory, represents a distance of separation of 5 nm [105]. Since cellular compartments are of much larger dimensions, fission follows a topological transformation of the limiting membrane into a highly curved tube-like intermediate (figure 18). The seminal discovery of reversible paralysis in flies harbouring a temperature-sensitive allele of the GTPase dynamin [106], which causes defects in the fission-induced birth of synaptic vesicles, prompted a surge in researching form and function of this molecule. Following this, purified dynamin was shown to form ring-like assemblies in solution and to be capable of vesiculating liposomes in the presence of GTP [107, 108]. Dynamin-catalyzed fission reaction has since been reconstituted on a variety of membrane templates using read-outs from light scattering, fluorescence microscopy and ion-conductance based approaches. Together, these efforts have unravelled three fundamental aspects about dynamin (figure 18): (a) dynamin binds and assembles on membranes containing anionic lipids into a 50 nm-wide helical scaffold that constricts the underlying tube to an intermediate with a 14 nm-wide lumen [11, 109]; (b) assembly triggers GTP hydrolysis that causes radial compaction of the scaffold to reach dimensions of 40 nm and forces the lumen to reach 4 nm. This facilitates the formation of the hemifusion intermediate wherein the inner monolayer of the tube is fused but the outer monolayer is separate [50, 110]; (c) interactions between the membrane-binding PHD in dynamin and anionic lipids facilitates rupture of the hemifusion intermediate thus completing the process of fission [48, 56]. As is the case with any scientifically active field, the community is still at debate on many aspects of membrane fission [54].

### Current and future challenges.

Here are some questions that remain unanswered with regards to dynamin in particular and membrane fission in general.

1. How are conformational changes in dynamin relayed to affect tube constriction and fission? GTPase activity causes a greater degree of constriction of the tube lumen compared to the dynamin scaffold indicating that a series of segmental conformational changes ultimately affects tube constriction and fission. Recent data from monitoring conformation of domain segments in different nucleotide-bound states and complemented by FRET and crosslinking strategies inform us of a powerstroke-like downward movement of the stalk upon GTP hydrolysis [48]. While these provide the first indications of a motor-like activity of this mechanozyme, the relative positions of the different domains of dynamin from the surface of the lipid bilayer during the GTPase cycle remains unknown and confounds interpretations on fission mechanisms. This is akin to the problem of deciphering if the earth moves around the sun or vice versa, which necessitated the mapping of motions relative to a third relatively-fixed celestial object. Furthermore, recent results [48, 56] indicating that the conversion of the hemifusion intermediate to full fission is not spontaneous but requires specific interactions between PHD and the membrane calls for a deeper, molecular-level understanding of lipid-protein interactions.
2. How universal is the dynamin paradigm? Dynamin follows a motor-like mechanism whereby the protein scaffold utilizes GTP hydrolysis to constrict the underlying tube for fission. This paradigm however is built solely on monitoring the behavior of classical dynamins. In fact, till date, fission has not been recreated in a reconstituted set-up even with the closely related mitochondrial homologs of dynamins. Furthermore, the molecular players and mechanisms by which fission is managed in numerous clathrin- and dynamin-independent vesicular transport pathways either remain a mystery or are at a nascent stage of understanding (see other articles in this series). Whether the characteristic self-sufficiency in function seen among classical dynamins is even present among other potential fission catalysts remains largely unknown. A discovery-based approach to catalog the molecular machinery involved in the myriad pathways of vesicle production in cells would therefore be required before we can truly appreciate the fundamental design principles of membrane fission.

### Advances in science and technology to meet challenges.

The questions raised above are by no means trivial and require a community-wide effort, drawing from expertise in biophysics, molecular dynamic simulations, and cell biology. To facilitate both a molecular-level understanding of dynamin function and the discovery of novel fission catalysts, we have developed a novel assay system of SMrT [11]. These templates represent an array of membrane nanotubes and a planar lipid bilayer pinned to passivated glass coverslips which together allows for fluorescence microscopy-based analysis of (a) membrane curvature-sensitive reactions; (b) reaction dynamics at the single fission event resolution; and (c) novel fission activity in cell lysates to expand the repertoire of fission catalysts. Given that these templates can be formed using standard resources available to most in the experimental community, we hope these assay systems would accelerate research into membrane fission.

### Concluding remarks.

While the past decade was dominated by structure-guided analyses of the workings of one fission catalyst, the coming years show the promise of vigorous research efforts into understanding dynamic aspects of membrane fission along with special emphasis on the discovery of novel fission catalysts. These developments should hopefully lay out a consensus mechanistic description of membrane fission, a process that is fundamental to life.

Acknowledgments

Research in the author’s laboratory is supported by grants from the Wellcome Trust-DBT India Alliance as well as the Howard Hughes Medical Institute. The author is an International Research Scholar of the Howard Hughes Medical Institute.

## Membrane remodeling by protein crowding

Wade F Zeno^1^ and Jeanne C Stachowiak^1,2^

^1^ Department of Biomedical Engineering, University of Texas at Austin, Austin, TX, United States of America

^2^ University of Texas at Austin, Institute for Cellular and Molecular Biology, University of Texas at Austin, Austin, TX, United States of America

### Status.

**Figure 19.**
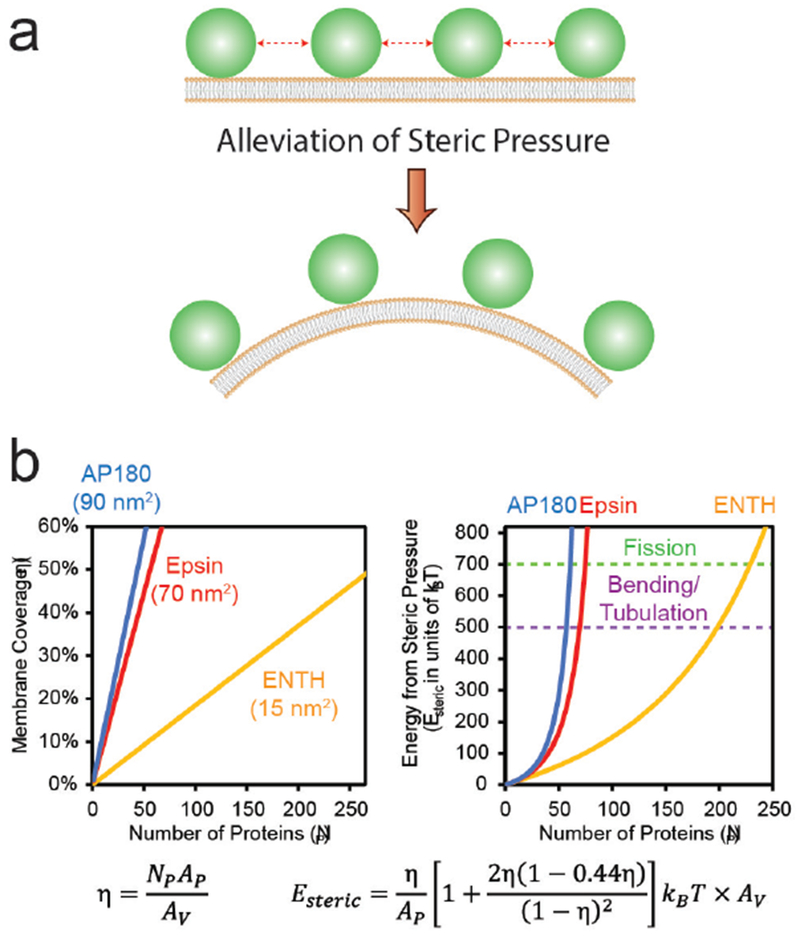
(a) Schematic of membrane induced bending via steric pressure that results from crowding on the membrane surface. (b) Membrane coverage (*η*) and the resulting energy from steric pressure (E_steric_) for representative proteins as a function of protein content on the membrane surface (N_P_). Plots were generated using the equations displayed, where E_steric_ is calculated using the Carnahan-Starling equation of state [113]. A_P_ represents the 2D protein area on the membrane surface, A_V_ corresponds to the surface area of the lipid vesicle, k_B_ is Boltzmann’s constant, and T is temperature. A vesicle with a diameter of 50 nm was used for these calculations.

Over the past two decades, the prevailing view has been that membrane curvature is controlled by the assembly of proteins with well-defined structural features including lattice-like spherical coats, tubular BAR-domain and ESCRT scaffolds, wedge-like amphipathic helices, and bundled cytoskeletal filaments. However, a persistent question has remained—how does the heterogeneous and highly crowded environment of the cellular membrane surface modify the function of protein assemblies? In the 1990s, biophysicists observed that the curvature of a membrane surface and its coverage by membrane-bound polymer molecules are inherently coupled. Specifically, Lipowsky *et al* demonstrated that whenever the density of polymer molecules on one membrane surface is higher than the density on the opposite membrane surface, the membrane takes on a curved shape, owing to the difference in steric pressure on the two membrane surfaces [111]. At a mechanistic level, the greater frequency of collisions among the polymer molecules on the more densely covered surface overcomes the opposing pressure on the less-densely coated side, leading to a pressure gradient that relaxes only when the membrane takes on a non-zero curvature. This concept is illustrated in figure 19, where the membrane bends to alleviate a build-up of steric pressure on the upper leaflet (figure 19(a)). Depending on the extent of the gradient, the resulting curvature can be quite high, an idea which has found industrial application in the ‘steric stabilization’ of liposomal drug carriers with diameters ranging from 30 to 70 nm.

**Figure 20.**
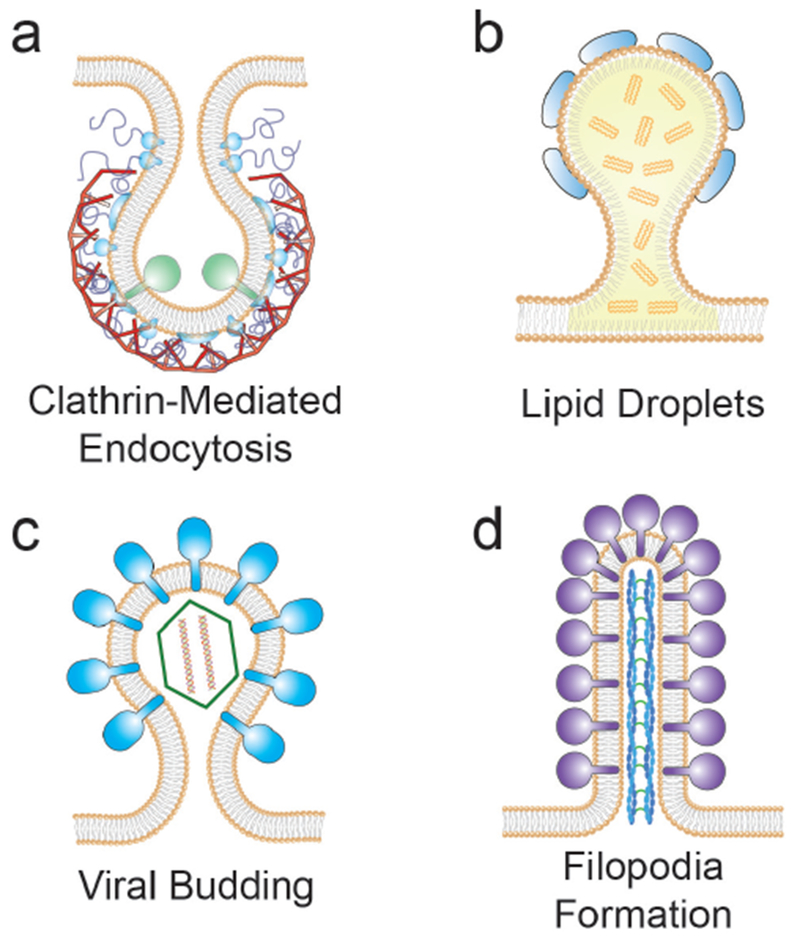
Representative scenarios in which membranes bend under crowded protein conditions. (a) Formation of a clathrin-coated vesicle during endocytosis as extracellular cargo is internalized, (b) formation of lipid droplets for lipid transport and storage, (c) formation of viral buds as viral receptors are overexpressed on the plasma membrane surface, and (d) assembly of filopodia at the plasma membranes of breast cancer cells is not only facilitated by bundled actin filaments, but also overexpression of receptor tyrosine kinases.

On the basis of these and other related findings, our laboratory asked whether membrane-bound proteins might also create steric pressure gradients capable of driving membrane shape changes. In early experiments, we demonstrated that locally concentrated patches of membrane-tethered globular proteins, including GFP, are capable of transforming membrane surfaces into tubules of high curvature [112]. Extending these findings to proteins involved in membrane shaping and endocytosis (figure 20(a)), we showed that the ability of the ENTH (epsin N-terminal homology) domain to drive formation of membrane tubules [113] and vesiculation of the membrane to form smaller vesicles [114], is correlated with the fractional coverage of the membrane surface by proteins (figure 19(b)), rather than with the insertion of amphipathic helices into membranes, as had previously been hypothesized. These observations have been applied and extended in diverse fields. A notable example is the demonstration by Walther *et al* that crowding among proteins that mediate lipid metabolism is required for the biogenesis of lipid droplets (figure 20(b)) [115]. Membrane bending by protein crowding can also be observed in viral budding. Overexpression of extracellular viral receptors aid in membrane vesiculation (figure 20(c)), which culminates with the release of enveloped viral particles.

If steric congestion promotes membrane curvature, then it is reasonable to assume that, on a per molecule basis, larger membrane-bound proteins will have greater impact on local steric pressure than smaller membrane-bound proteins. Interestingly, many of the proteins involved in membrane traffic have long been known to contain bulky, intrinsically disordered domains, which occupy considerably larger volumes in comparison to structured motifs of equivalent molecular weight. Therefore, our recent work has investigated the role of intrinsically disordered domains in generating steric pressure at membrane surfaces, demonstrating that these domains, which lack a stable structure, are in fact among the most potent drivers of membrane vesiculation [116] and fission [114]. This concept is illustrated in figure 19(b), in which three different endocytic proteins are compared in terms of the area they occupy per molecule on the membrane surface and their capacity to generate steric pressure. AP180 and Epsin, which each contain large intrinsically disordered domains, are much more effective at crowding the membrane surface and driving membrane remodeling in comparison to the small globular ENTH domain.

Importantly, the luminal face of a curved membrane structure, which must take on a concave morphology during membrane vesiculation, is also typically highly crowded by membrane-bound protein domains such as the ectodomains of transmembrane cargo proteins, among other constituents. The resulting steric pressure on this surface would be expected to oppose membrane vesiculation. How might this steric opposition influence membrane traffic? The work from Silvius *et al* demonstrates that steric pressure plays an important role in helping endocytic pathways sort cargo molecule content [117]. Similarly, the work by Miller *et al* suggests that bulky or asymmetrically shaped transmembrane cargo molecules generate steric pressure that opposes their incorporation into trafficking vesicles, and therefore require a denser, more mechanically rigid coat for ER export [118]. Finally, the work from Sliwkowski *et al* suggests that when receptor tyrosine kinases reach extremely high expression levels on the surfaces of breast cancer cells, the resulting steric pressure can help promote outward buckling of the membrane during filopodia formation (figure 20(d)) [119]. Collectively, this body of work suggests that membrane shape, both *in vitro* and in living cells, is strongly influenced by the balance of steric pressure between opposing membrane surfaces.

### Current and future challenges.

While steric pressure has been implicated in a number of essential cellular processes, it remains a significant challenge to directly measure and characterize the impact of protein crowding in live cells. In particular, it is often difficult to differentiate between the biophysical and biochemical contributions made by specific protein constituents. For example, the clathrin coat is recruited by a family of adaptor proteins that form an interconnected network that links lipids and cargo proteins to the clathrin lattice (figure 20(a)). While individual adaptor proteins have been demonstrated to generate substantial steric pressure *in vitro* [113, 114], the situation within the cell may be much more complicated. Specifically, it remains unknown how the assembly of interconnected protein networks modulates steric pressure. On the one hand, the ability of a protein network to locally concentrate proteins may increase steric pressure among some network partners. On the other hand, condensation of proteins into a network may reduce their conformational entropy and freedom of motion, leading to a reduction in steric pressure. A related challenge emerges from the diversity of potential curvature driving proteins and mechanisms. In particular, it is important to understand how diverse mechanisms may collaborate to shape membrane surfaces. Such collaboration may arise from the presence of distinct curvature driving motifs within the protein network, or even within the same molecule. For example, the clathrin adaptor amphiphysin contains three potential curvature drivers: a curved BAR domain, a membrane-inserting amphipathic helix (AH), and a bulky disordered domain, which may be capable of generating steric pressure. Resolving these complex interactions requires the study of full-length proteins and protein ensembles rather than individual domains, as well as the development of creative strategies for dissecting biophysical mechanisms in live cell experiments.

### Advances in science and technology to meet challenges.

To address the challenges highlighted above, several specific advances are needed. From an experimental perspective, assays for measuring steric pressure at membrane surfaces both *in vitro* and in live cells must be developed. Methods relying on FRET-based measurement of the extension of polymer chains and protein-based spring-like domains have been previously applied to measure entropic pressure in crowded solutions [120]. Similar techniques could be adapted to study steric pressure at membrane surfaces. Additionally, experiments should be initiated to carefully quantify the molecular stoichiometry of curved membrane structures at multiple stages of assembly. The increasing availability of gene editing techniques and super-resolution imaging approaches puts this goal increasingly within reach. Finally, from the theoretical perspective, the development of models that can simulate the collaboration between multiple curvature driving mechanisms represents a gap in the existing literature. With particular regards to the steric pressure mechanism, greater understanding of the pressure-coverage relationship for both globular proteins and intrinsically disordered domains is needed in order to more accurately predict the magnitude of steric pressure based on measurements of molecular stoichiometry.

### Concluding remarks.

While it was originally thought that proteins shape membranes exclusively through the use of specific structural motifs, it is now understood that an imbalance of steric pressure on the two sides of a membrane surface can provide a potent driving force for membrane curvature and even membrane fission. Importantly, any protein, regardless of structure, can utilize this mechanism to generate substantial steric pressure when sufficiently concentrated on membrane surfaces. Paradoxically, protein that lack a well-defined structure, intrinsically disordered domains, may ultimately be the most efficient generators of steric pressure owing to their large volume to mass ratio. The emerging understanding of this non-specific mechanism of membrane curvature generation suggests that membrane shape may be influenced by a much larger group of protein species than previously thought, many of which likely remain to be discovered. Progress in this exciting field depends upon improved methods for measuring and observing the effects of steric pressure both *in vitro* and in cells, as well as the development of models that accurately integrate the synergistic collaboration between multiple curvature driving mechanisms.

Acknowledgments

The authors acknowledge research support for the US National Institutes of Health under Grant R01GM120549 to Stachowiak.

## Sensing membrane curvature

Dimitrios Stamou, Artù Breuer and Line Lauritsen

Bionanotechnology and Nanomedicine Laboratory, Department of Chemistry, Nano-Science Center, University of Copenhagen, Copenhagen, Denmark

### Status.

The membranes of cells and cellular organelles adopt 3D shapes which are highly conserved, suggesting membrane shape is crucial for life [121]. To date, we understand a lot about how membrane shapes are induced and maintained, however we know relatively little about the implications that different membrane shapes may have on biological function once they are created. Historically, this has been largely due to the absence of methods to investigate the effects of membrane shape but also due to lack of awareness of the functional significance of membrane shape. Over the last decade, however, we have witnessed a proliferation of such methods and investigations that have collectively helped establish the notion that membrane shape is not a passive side-effect of other biological processes, like for example membrane trafficking, but instead a causal, bona fide, regulator of multiple disparate biological functions (see [122] and all citations therein).

**Figure 21.**
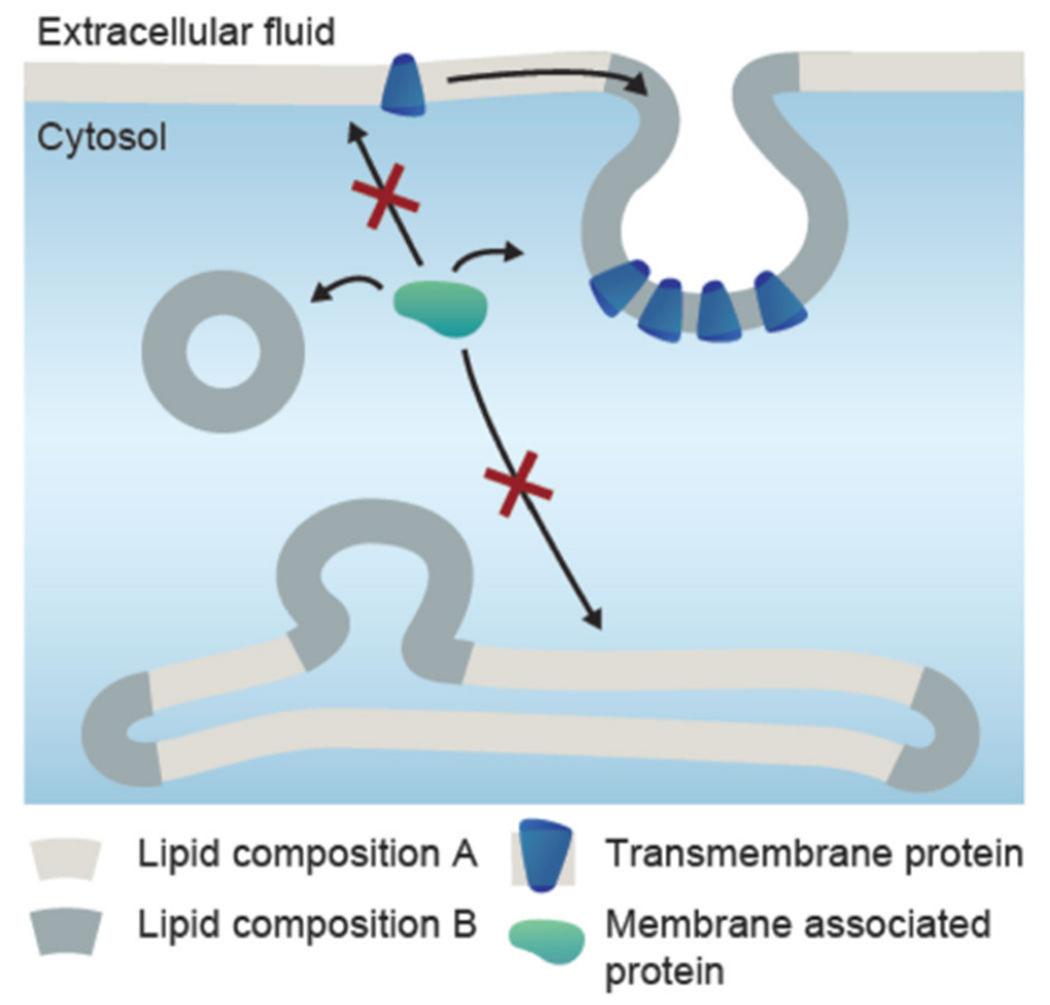
Curvature modulates the packing of lipid headgroups and acyl chains and thus the mesoscopic physical properties of membranes. Consequently, membrane curvature can act as regulatory cue that modulates the localization of lipids, membrane-associated and transmembrane proteins.

**Figure 22.**
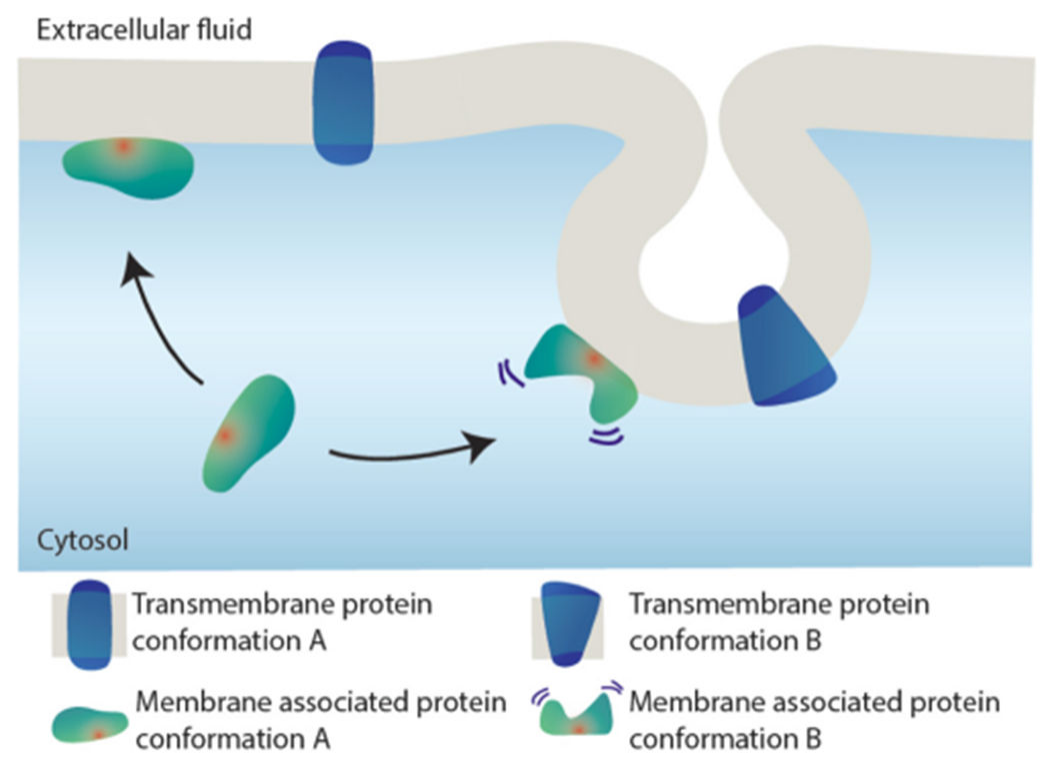
Membrane curvature can also modulate the structure/conformation and function of both membrane-associated and transmembrane proteins, including protein kinase C, membrane spanning pores and G protein coupled receptors. These findings broaden tremendously the scope and implications of membrane curvature sensing.

The term ‘sensing’ of membrane shape was originally coined to denote preferential spatiotemporal localization of proteins in membranes of high local curvature; figure 21 [2]. BAR domains [2] and amphipathic helices [123] are the most studied structural motifs that bind peripherally to membranes and can sense membrane curvature. However, subsequent experiments revealed that the physical properties of curved membranes can also modulate the localization of lipids [124], lipidated proteins [125] and transmembrane proteins [126]. Importantly, we now have the first indications that membrane curvature can affect not just the localization but also the structure and function of both peripheral and transmembrane proteins, including protein kinase C, membrane spanning pores [127] and G protein coupled receptors [126]; figure 22. The latter findings broaden tremendously the scope and implications of membrane curvature sensing.

Cells are composed of a myriad of individual molecules (proteins, nucleic acids, lipids, etc) whose structure, chemical identity, and spatial and temporal organization are in constant flux as part of multiple, often coupled, dynamic equilibria. Membrane shape has only recently emerged as a pluripotent regulatory hub in this labyrinthine network of interactions. Work done over the coming decade should focus on clarifying its relative importance amongst the existing repertoire of chemical and physical regulators of cellular membrane biology [122].

### Current and future challenges.

In our opinion, the field is facing two main challenges in the foreseeable future. The first one can be summed up with the words ‘not all curvatures are the same’. The highly-conserved assignment of different membrane shapes to different organelles (see the Golgi apparatus versus the ER) suggests distinct functions are assigned to distinct shapes. However, because of the great difficulty in developing membrane curvature assays, the majority of quantitative investigations in reconstitution have used either liposomes or membrane tubes as proxies of high membrane curvature, implicitly assuming that spherical and cylindrical curvatures are equivalent [128]. This ambiguity can be resolved via a side-by-side quantitative experimental and theoretical comparison of membranes with different geometry, and thus mean and Gaussian curvatures. Such investigations should provide critically essential insights that will help us rationalize why specific membrane shapes emerged through evolution and why they were conserved.

The second main challenge is connecting in a more rigorous manner cellular phenotypes with the insights already reached through theory, simulations and experiments in reconstitution. A few selected quantitative live-cell studies have embarked already on this route [126, 129, 130]. Their contribution is of great importance because they constitute proofs of concept that selected membrane curvature driven processes are not outcompeted but are, on the contrary, significant enough to play a role in the complex cellular environment. Further investigations in this direction will be essential to take the field beyond these proofs of concept.

### Advances in science and technology to meet challenges.

The aforementioned challenges can in principle be met by exploiting existing technologies. Two relevant examples are the very recent Nobel Prizes in Chemistry that were awarded for technologies able to resolve the distribution of proteins and lipids within cells with high spatial resolution, super-resolution and cryo-electron microscopy in 2014 and 2017, respectively. Additionally, of course, fluorescence microscopy can be used to report on molecular structure and/or function. Given the sophistication of existing technologies in place today, it is more a question of selectively and purposefully applying them to unravel the contribution of membrane shape to molecular organization, structure and function.

### Concluding remarks.

The living cell comprises a sophisticated, hierarchical, integration of spatiotemporal variations in membrane composition and shape, with multiple other bio-physico-chemical signalling cues. We should strive towards correlating these diverse parameters within the actual environment of living cells using next generation super-resolution high content analysis. Such studies will eventually yield an integrated view of cell biology spanning multiple scales of length and levels of organisation [122].

Acknowledgments

D S would like to express his deepest gratitude to all the postdocs, PhD, MSc and BSc students that have worked in the lab on this topic. This work was supported by the Lundbeck Foundation in the past, and currently by the Innovation Fund Denmark (5184-00048B) and a Novo Nordisk Foundation grant for the Center for Geometrically Engineered Cellular Systems (NNF17OC0028176).

## Cytoskeleton-mediated membrane reshaping in endocytosis

Camille Simon^1,2^ and Cécile Sykes^1,2^

^1^ Laboratoire Physico Chimie Curie, Institut Curie, PSL Research University, CNRS UMR168, 75005, Paris, France

^2^ Sorbonne Universités, UPMC Univ Paris 06, 75005, Paris, France

### Status.

**Figure 23.**
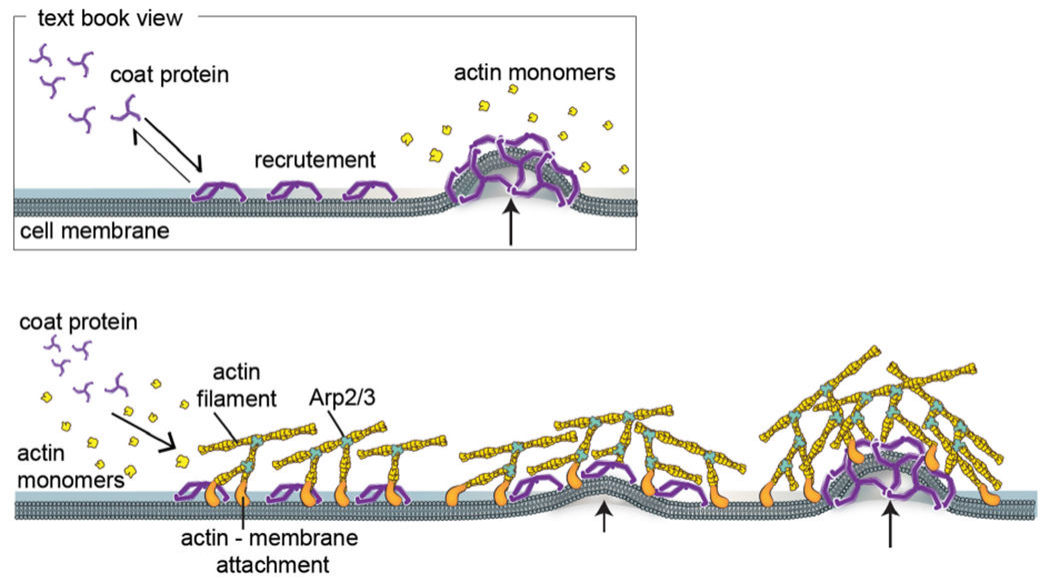
Schemes for early stage of endocytosis: newly evidenced chronology of membrane bending by actin dynamics that may further assemble coat proteins. Inset: original textbook view: coat proteins first bend the membrane by spontaneously assembling.

How membranes change shape during endocytosis has fascinated biochemists, biologists and physicists in the last twenty years. During endocytosis, the plasma membrane first deforms inward or ‘buds’, then the bud elongates and the membrane deformation appears tube-shaped before it breaks into a vesicle. Observation of budding, and of the whole process of endocytosis is difficult because the size of the endocytic intermediates is below the resolution limit of light microscopy. Initial budding has been first believed to entirely rely on coat-protein assembly (figure 23, inset), and *in vitro* reconstitution systems using pure membranes and purified coat proteins were able to produce curved membranes [131]. Therefore, this view became the textbook view. However, a recent careful electron microscopy study correlated with fluorescence microscopy revealed that this is not the case, at least unambiguously in yeast [132] and in mammalian cells under certain conditions [133]. Indeed, when coat proteins are recruited to the plasma membrane, the surface of the membrane remains unmistakeably flat and does not deform (figure 23). Membrane deformation into a bud only appears when actin polymerization is triggered at the (flat) membrane (figure 23). Conversely, in conditions where cells are treated with actin depolymerizing agents, the membrane never deforms and remains flat, although coat proteins and most molecules of the endocytic machinery are correctly recruited. Therefore, actin dynamics and its assembly into growing filaments is the main player of membrane deformation during the early stage of endocytosis. Moreover, it is now established that the growing actin network involves the Arp2/3 complex machinery that elongates actin filaments through side branching [134]. However, the physical mechanism that generates membrane budding through actin filaments that branch at the membrane surface is still poorly understood. It may involve membrane tension and protein concentration. However, the minimal actin network structure that is able to pull on the membrane is not yet known. Simplified reconstitution systems, high resolution imaging technologies, and cell manipulations will help to address these questions.

### Current and future challenges.

One of the main challenges for the study of endocytosis is the size of membrane deformations, a few tens of nanometers, not easily accessible by optical microscopy [132]. Moreover, endocytosis occurs within about 10 s [132]. Impressive correlation methods have been developed to overcome this challenge and combine fluorescence imaging either with high resolution in time, or with high resolution in space using electron microscopy imaging in cells [132, 135, 136]. Membrane invaginations correlate with the growth of a branched, dynamic actin network at the site of endocytosis. Branches are dynamically formed at the membrane [137]. Membrane bending through actin dynamics is only possible if (1) the force exerted by network growth is above a certain threshold and (2) there is a physical link between the membrane and the network [138]. This branched network, extensively studied *in vitro* as a continuous viscoelastic network, has a mesh size of 30–50 nm, about the same size as the width of a tubular endocytic invagination. Therefore, the pull-out of the membrane relies only on a few filaments that are part of a more extended actin network. One of the challenges is to understand the role of these few filaments that are bound to the membrane strongly enough to exert a point pulling force, and part of a wider spread actin network that grows from the membrane around this point. The existence of this surrounding outward-growing network is evidenced by its filtering effect of size-excluded ribosomes that are absent from an ‘exclusion zone’ of about 200 nm around the endocytic site. Interestingly, another effect of a growing actin network, which polymerization is triggered at a membrane, can be that it concentrates, by convection, proteins at the surface [139], as it may be the case for clathrin (see the scheme in figure 23). Finally, cross linkers that change the mesh size of the network and its rigidity, are shown to be crucial for the formation of the invagination [138]. How cross-linkers can change the force needed to pull out the membrane is to be understood.

### Advances in science and technology to meet challenges.

**Figure 24.**
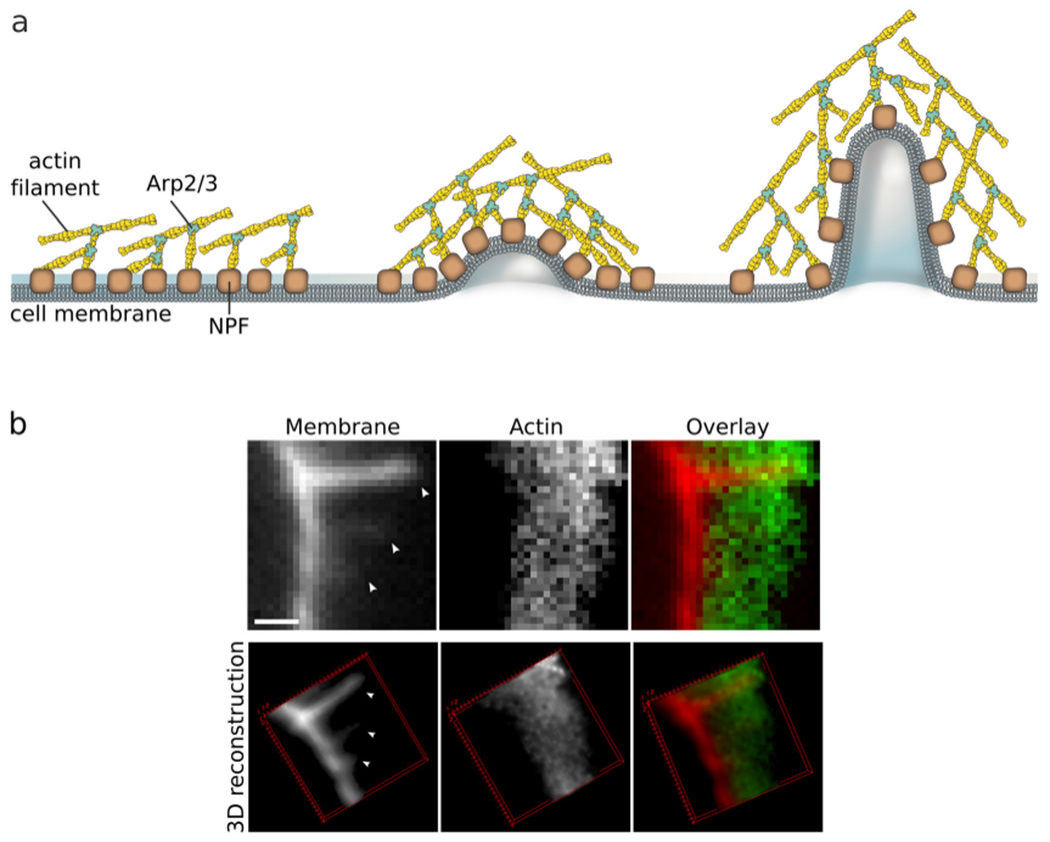
*In vitro* reconstitution of membrane bending and tubule growth by sole actin dynamics. (a) Scheme of the experiments. (b) Confocal images of the membrane and actin, and 3D reconstructions. Scale bars, 1 *μ*m.

Detecting the early stage of membrane invagination is still a challenge, in cells, and also in *in vitro* systems that may reproduce some stages of endocytosis. A technological advance here may be to design curvature sensors that would, for example, emit a change in fluorescence signal. *In vitro* systems are of choice to address how (and if) a (simple) dynamically branching network is able to drive membrane deformations at the scale of endocytosis. A pure membrane can be designed to recruit a nucleation promoting factor (NPF) that triggers the assembly of fluorescent actin monomers into branches [140]. The incorporation of fluorophores in the membrane allows its direct visualization. Such systems, using pure membranes and the dynamic machinery of actin with the branching agent, the Arp2/3 complex, already provide the scientific proof that a membrane can be tubulated in these conditions (figure 24, and unpublished results). Time imaging of the membrane deformation and its surrounding will allow to follow the early stage of membrane invagination. Bending and the subsequent growth of the plasma membrane invagination require the plasma membrane to be physically linked to the actin network. Attachment of the actin cytoskeleton with the membrane is here provided by the NPF (that activates the Arp2/3 complex) bound to the membrane with a biotin-streptavidin link (figure 24), and is sufficient to provide membrane pulling. Such systems, with the advantage that the attachment of the cytoskeleton to the membrane can be tuned, can be further complexified with the addition of cross linkers for example. Membrane tension may affect the initial membrane invagination for endocytosis, as suggested in the complex context of mammalian cells [133]. This aspect can also be addressed with *in vitro* systems by the use of a micropipette aspiration technique where membrane tension is controlled.

### Concluding remarks.

It appears increasingly clear that endocytosis relies on a fine tuning of local activation and orchestration of actin dynamics [135]. A way to prove it is to reconstitute endocytic-like deformations *in vitro*, in a minimal system that activates actin dynamics at a membrane. In general, *in vitro* systems provide powerful tools to address the exclusive role of specific proteins, or physical parameters, in cell functions. Nevertheless, the ‘simplicity’ of reconstituted systems needs to be constantly paralleled to the complexity of cellular systems, so that we gain a complete understanding of cell mechanisms and functions. Since more than 30 proteins participate to the job in cells [134], the precise chronology of the mechanism of endocytosis will be established with further back and forth studies of simplified, controlled systems, and whole cell systems.

Acknowledgments

We acknowledge Dr Agnieszka Kawska at IlluScientia.com for the figures. This work was supported by the French Agence Nationale pour la Recherche (ANR), Grant ANR-14-CE090006 and ANR-12-BSV5001401, and by the Fondation pour la Recherche Médicale (FRM), Grant DEQ20120323737. We thank Andrea Picco for usefull discussions on recent results on the endocytic machinery.

## Simulations of N-BAR protein interactions with membranes

Gregory A Voth

Department of Chemistry, James Franck Institute, and Institute for Biophysical Dynamics, The University of Chicago, Chicago, IL, United States of America

### Status.

**Figure 25.**
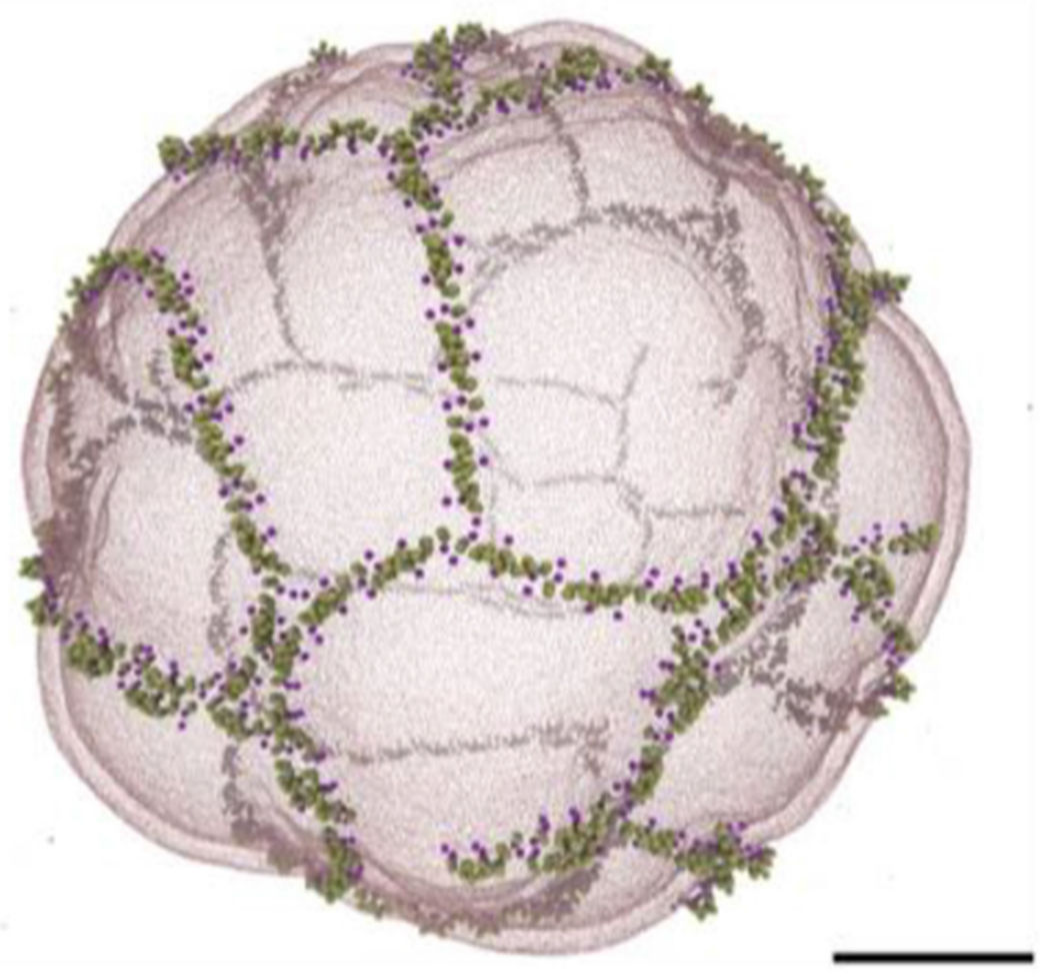
A CG MD simulation prediction of the linear aggregation assembly of N-BAR proteins on a lipid membrane leading to local vesicle deformations. Scale bar: 50 nm.

Membrane remodeling processes are innately multiscale, as they span the molecular to nanoscopic to mesoscopic time and length scales. For instance, the molecular-level interactions between collections of proteins and the lipid bilayer can have a profound effect on the large-scale membrane morphology [1]. Molecular models and multiscale simulation methods that combine all-atom (AA) and CG MD are aimed at describing these larger-scale processes. The development of CG simulation methods [1] has given us the ability to study membrane systems with bound proteins at time and length scales accessible, e.g. to optical and electron microscopy— and at scales much larger than AA molecular dynamics can reach alone. These methods have been applied with great success, for example, to elucidate the complex behavior of Bin/Amphiphysin/Rvs (BAR) proteins, which are key curvature regulators in the cell and are found in a number of curvature-related phenomena. These simulations have identified the way these proteins sense membranes at the molecular level and the mechanism by which they form macromolecular assemblies at the mesoscopic level (see, e.g. [141–143]) For example, using highly CG modeling, it has been discovered that, at low bound densities, N-BAR proteins can assemble into long strings or filaments that form a meshwork prior to budding of the membrane (see figure 25). Interestingly, these interactions among the proteins are driven purely by membrane curvature fluctuations and act at remarkably long ranges—an order of magnitude greater than typical screened electrostatic interactions [142]. Moreover, subsequent CG simulations showed that membrane tension can modulate these protein-protein interactions [143]. With the aid of simulation (especially CG simulation), it is thus becoming increasingly clear that N-BAR domain proteins have a diverse range of function related to endocytosis and other cellular processes, and the way in which they behave on the membrane largely depends on the protein surface density. Recent experimental studies have even shown that such multimodular activity of N-BAR proteins is crucial for the cell, including a very surprising finding that endophilin (an N-BAR protein) drives its own endocytotic pathway [9]. These discoveries have opened many significant questions for future exploration.

### Current and future challenges.

Indeed, armed with continually developing multiscale computational methods and our evolving understanding of protein-membrane interactions, there are at least three pressing future challenges in studying these N-BAR systems as follows: (1) BAR proteins peripherally adhere to the membrane and, in the case of N-BARs, insert an N-terminal AH into the bilayer. It has been shown than an isolated AH is unfolded in solution but folds when inserting into lipid packing defects on the membrane, and that such defects are more present on bent membranes with positive curvature [144]. The precise molecular mechanism by with the N-terminal amphipathic helices on the N-BAR domains sense and/or generate curvature is still under active debate and in need of resolution [6, 145]. (2) Even more complex multi-BAR protein interactions on the membrane surface must begin to be studied, including for the full length proteins such as endophilin and amphiphysin. This challenge comes in light of the conflicting experimental reports on the roles of simple protein crowding on membranes versus key structural motifs in generating membrane remodeling [113, 146]. (3) The important interaction between dynamin and N-BAR proteins must begin to be explored. All of these three challenges should also be addressed through a combined simulation/experimental effort.

To address the first challenge, next generation CG models (see the next section) can be utilized to elucidate how N-BAR proteins balance scaffolding and AH insertion mechanisms to control membrane morphology. The second challenge will be to scale up these CG models to study the assembly of many BAR proteins on the membrane and to delineate the role of crowding and how the N-BAR (and their AHs) appear to provide effects beyond crowding alone in driving membrane morphology [146]. An interesting aspect of the BAR domain is that it displays high variability in size and intrinsic curvature among different BAR proteins. Simulating multiple BAR proteins on the same membrane will show how they assemble and generate curvature, whether they cooperate with one another, and if the association of highly curved BARs requires the assembly of the lower curved BARs first. Working with experimentalists, this challenge can be addressed by exploring N-BAR protein-protein interactions relevant to endocytosis at a higher level of complexity, by including multiple types of proteins in reconstituted *in vitro* and *in silico* systems. This work will help to unravel a very challenging question: how different endocytic proteins ‘find one another’ at the right place and at the right time. The mystery surrounding this issue is especially evident in the case of sorting nexins—a family of BAR proteins—whose recruitment is precisely controlled in space and time during endosomal trafficking, a process taking place after endocytosis.

The third challenge will involve an investigation the way N-BAR proteins interact with dynamin on membrane tubules. Recent experiments have shown that N-BARs directly recruit dynamin to the membrane and that their presence is crucial for efficient dynamin-mediated fission in endocytosis [147–150]. Although cryo-electron microscopy combined with CG simulations have elucidated how N-BARs assemble on certain membrane tubes, these experiments explored only a very high packing density (>90% coverage) of the protein on the surface, which cannot permit the binding of dynamin. An understanding of N-BAR protein structure and dynamics on membrane tubules at more relevant physiological surface densities is therefore required to elucidate how they take part in endocytosis. CG MD simulations [6] have in fact observed that N-BARs can readily assemble on membrane tubules, much like on a quasi-flat surface of the vesicle. Interestingly, the proteins form a scaffold on the tubule at the surface density of ~30%, as has been inferred from fluorescence microscopy under similar near-physiological conditions done in the Bassereau Lab. In the future, it will be thus be key to investigate how the full-length N-BAR protein endophilin affects the binding of dynamin on the tubule and in turn controls membrane fission.

### Advances in science and technology to meet challenges.

At least two important advances in simulation methodology must be utilized and expanded to meet the challenges of the N-BAR protein simulations elaborated in the previous section. In the area of AA MD simulation, recent significant extensions of the metadynamics (MetaD) enhanced free energy sampling approach will allow for a much greater exploration of the molecular-scale interactions responsible for the N-BAR to membrane and N-BAR to N-BAR associations. The MetaD method is both exact and convergent, and it has also led to the development of new and more powerful MetaD approaches, called transition tempered MetaD (TTMetaD) and meta-basin MetaD (MBMetaD). The latter is especially important since it allows for the targeted free energy sampling of the conformational landscape in specific regions of proteins and membranes. In turn, TTMetaD accelerates the convergence of the sampling in a demonstrably substantial way, and the two new MetaD approaches can be used together. Since MetaD enhances the sampling along collective variables, it will be ideal to utilize insight gained from CG models which are developed in terms of coarse variables and thus more readily projected onto collective variables. Such an approach can also provide additional detailed information on the molecular-scale interactions ‘underneath’ the CG models and, in turn, to help refine these models.

**Figure 26.**
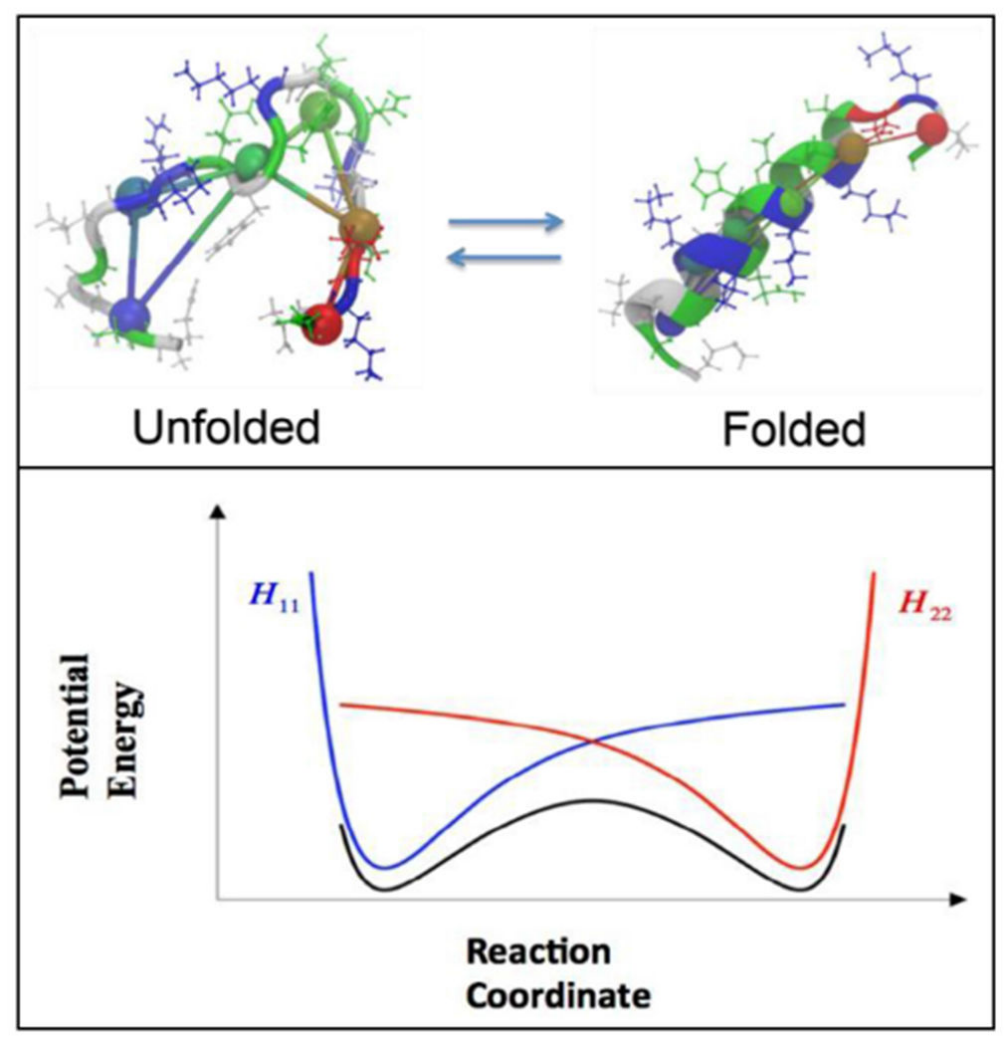
(Top) A highly CG model of the AH of endophilin is depicted. (Bottom) The MCCG method allows transitions between conformational states on the fly via two coupled diabatic CG states *H*_11_ and *H*_22_ (red and blue) coupled through a 2 × 2 matrix with coupling elements *H*_12_, for the conformationally mixed MCCG state (black).

A new ‘multi-configurational’ coarse-grained (MCCG) approach can also be developed to describe explicit large scale protein conformation change at the CG level (see figure 26 for the case of the N-BAR AH folding). A key aspect of the overall approach for the CG models will be to ‘divide and conquer’ the coarse-graining problem for the effective CG potentials (see figure 26, bottom) and to represent the conformational change proce,ss by an ‘on the fly’ 2 × 2 matrix solution (or even more matrix ‘states’ as needed). The more physically accurate MCCG model can, for example, represent the N-BAR AH folding process as N-BARs are in the process of binding to the membrane and associating with each other.

### Concluding remarks.

While a great deal has already been accomplished in the simulation of N-BAR proteins and their role in membrane remodeling, much remains to be done to more fully elucidate their complex and varied roles in endocytosis and other cellular processes. In turn, these challenges will require new breakthroughs in both AA and CG simulation methodology—as well as a multiscale bridging of the two—and the development of additional connections with ground-breaking experimental results.

Acknowledgments

The research reported in this publication was supported by the National Institute of General Medical Sciences of the National Institutes of Health under award number R01-GM063796.

## Membrane-mediated interactions of nanoparticles

Thomas R Weikl

Department of Theory and Bio-Systems, Max Planck Institute of Colloids and Interfaces, Science Park Golm, 14424 Potsdam, Germany

### Status.

**Figure 27.**
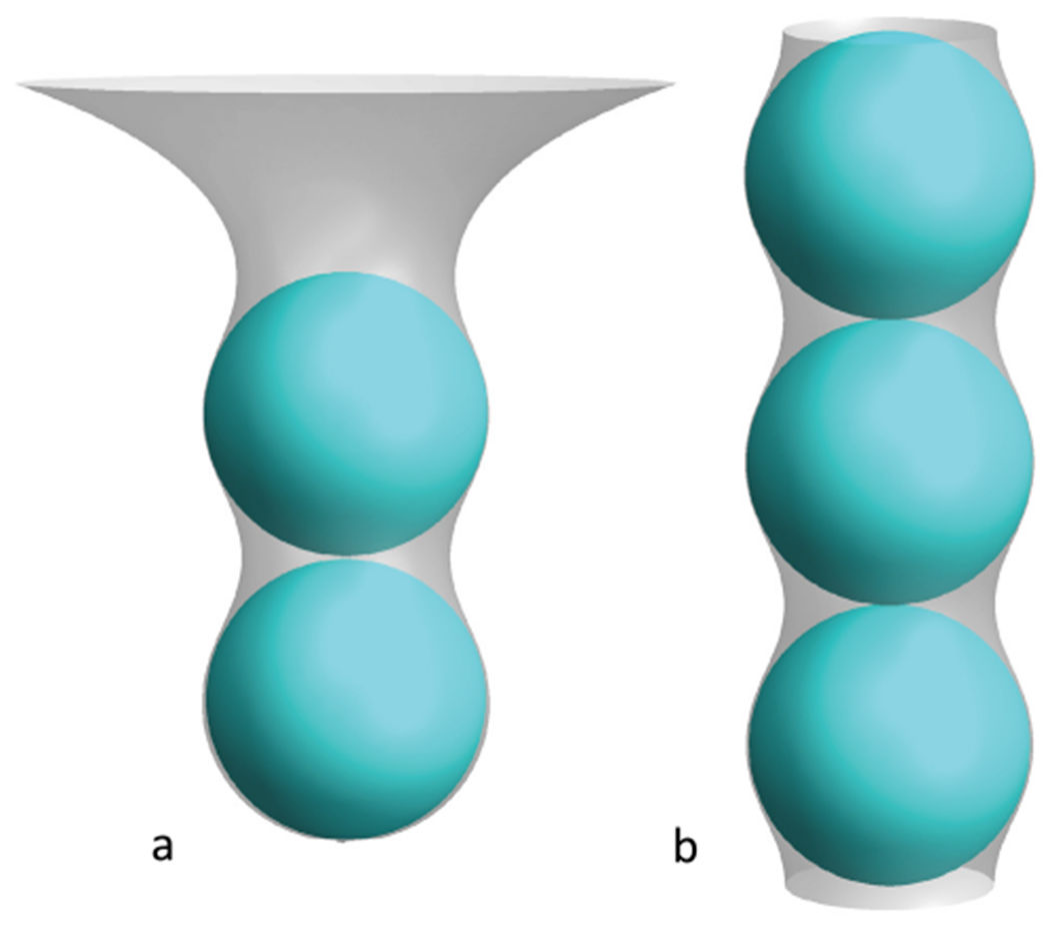
(a) Minimum-energy conformation of two spherical particles in a membrane tube that is connected to a planar membrane via a catenoidal membrane neck. (b) Three central spherical particles in a membrane tube. Reproduced from [153] with permission of The Royal Society of Chemistry.

The adsorption of spherical or ellipsoidal nanoparticles to membranes induces a local membrane curvature, which leads to indirect membrane-mediated interactions between the nanoparticles [151, 152]. These indirect interactions can be strong and can dominate over direct interactions such as van der Waals or electrostatic forces that govern the assembly of nanoparticles in solution [153]. The indirect, membrane-mediated interactions of adsorbed nanoparticles result from the fact that the total energy of membrane bending and particle adhesion depends on the distance of the particles. Simulations and experiments indicate the attraction of two membrane-adsorbed spherical particles if the distance between the particle surfaces is smaller than the particle diameter [151, 152]. In their minimum-energy state, the two particles are in close contact and are cooperatively wrapped by a membrane tube in which one of the particles sits on top of the other particle (see figure 27(a)). Membrane tubes that cooperatively wrap several particles are energetically favorable and show a periodicity in which bound membrane segments that smoothly wrap the particles alternate with unbound membrane segments between the particles (see figure 27(b)). These unbound membrane segments adopt the shape of a catenoidal minimal surface with zero mean curvature and, thus, zero bending energy. For spherical particles, the energy gain of the cooperative wrapping strongly depends on the interaction range of the particle-membrane adhesion potential and results from a favorable interplay of bending and adhesion energies in the boundary regions between the bound and unbound membrane segments. In these boundary regions, the membrane ‘swings’ into the catenoidal shape of the unbound segments with zero bending energy but still gains adhesion energy. The cooperative wrapping is favorable because a central particle in a membrane tube has two such boundary regions while a single wrapped particle only has one boundary region [153].

**Figure 28.**
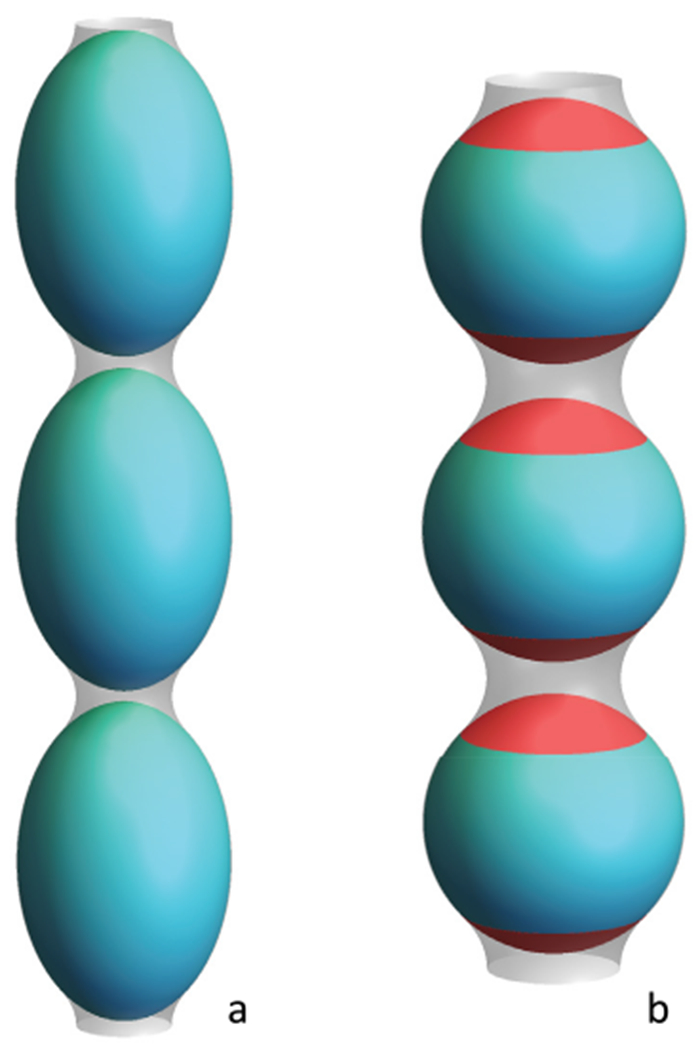
Minimum-energy conformations of (a) prolate particles and (b) triblock Janus particles in membrane tubes. The triblock Janus particles have non-adhesive tips (red) and adhesive sides (blue). [154] John Wiley & Sons. © 2016 WILEY-VCH GmbH & Co. KGaA, Weinheim.

In contrast to spherical particles, the cooperative wrapping of elongated, prolate particles in membrane tubes is energetically highly favorable for all interaction ranges of the particle-membrane adhesion potential [154]. Prolate particles have a rather high mean curvature at their tips and a lower mean curvature at their sides. Membrane adhesion to the tips of prolate particles therefore costs more bending energy than adhesion to the sides. The cooperative wrapping of the prolate particles in the membrane tube of figure 28(a) is energetically favorable compared to the individual wrapping of the particles because the tubular membrane only adheres to the sides of the prolates and not to the tips. Similarly, the cooperative wrapping of the triblock Janus particles of figure 28(b) is energetically favorable because the non-adhesive tips (red) remain unwrapped, which saves bending energy compared to the individual wrapping of the particles in which at least one of the tips needs to be wrapped. For all these particles, the energetically favorable cooperative wrapping in tubes implies attractive membrane-mediated interactions.

### Current and future challenges.

Membrane-mediated interactions between nano-objects such as particles and proteins have been studied intensively for more than 20 years and continue to be a current focus of research because these interactions can be the dominant driving forces for assembly and cooperativity. In classical elastic-membrane models, the nano-objects are taken to constrain the membrane curvature along a contact line. Rotationally symmetric objects such as conical transmembrane proteins and cap-shaped protein scaffolds or particles, for example, are taken to impose a fixed contact angle on the membrane along a circular contact line. The membrane-mediated interactions of nano-objects with circularly symmetric contact lines are overall repulsive in planar membranes [155] because the membrane bending energy is zero and, thus, minimal at large distances at which the membrane adopts a catenoidal shape of zero mean curvature around each of these objects. For crescent-shaped scaffold proteins such as BAR domains, in contrast, the contact line with the membrane is highly anisotropic, and the membrane-mediated interactions are strongly attractive if the proteins face each other side by side [156]. For the adsorbed spherical or prolate nanoparticles of figures 27 and 28(a), the contact line, i.e. the boundary region between bound and unbound membrane segments, is not fixed but results from the interplay of bending and adhesion energies, which leads to the membrane-mediated attraction and cooperative wrapping.

Besides these indirect interactions of nanoparticles and proteins that are caused by induced changes of the membrane curvature, membrane-mediated interactions and cooperativity can also result from a suppression of membrane shape fluctuations. Based on CG simulations and experiments, the aggregation of Shiga toxin proteins on membranes has been recently attributed to the suppression of membrane shape fluctuations caused by the tight binding of the rather large proteins to the membranes [77]. The suppression of membrane shape fluctuations by receptor-ligand complexes in membrane adhesion leads to cooperative binding because the binding constants of the complexes strongly depend on the nanoscale roughness of the membranes from shape fluctuations, which in turn depends on the concentration of the complexes [157]. Initial receptor-ligand complexes smoothen the adhering membranes and facilitate the formation of additional complexes.

A current and future challenge is to go beyond the classical elastic-membrane models and to include the molecular detail of membranes, proteins and nanoparticles in simulations of membrane-mediated interactions. Central questions are: can the curvature constraints along the contact lines of proteins and nanoparticles in elastic surface models be derived from molecular simulations in multi-scale modeling approaches? What is the role of specifically bound lipids for these curvature constraints, such as the lipids bound in binding sites of scaffolding proteins or in the lipid annulus around transmembrane proteins? And how does the strength and range of the adhesive interaction between nanoparticles and membranes depend on nanoparticle chemistry and membrane composition?

### Advances in science and technology to meet challenges.

Advances in simulation technology and methods will continue to increase the range of length and time scales accessible in CG and atomistic simulations of membranes interacting with nanoparticles and proteins. In recent years, the use of graphics processing units for atomistic simulations has clearly extended these ranges. Advances in nanoparticle synthesis may help to investigate the membrane-mediated interactions of adsorbed nanoparticles, or to mimic the scaffolding of proteins. For example, the cooperative wrapping in membrane tubes has been experimentally observed for spherical simian virus 40 particles [76] but not for synthetic nanoparticles. For spherical nanoparticles, the complete wrapping of single particles can be fast for sufficiently large adhesion energies of the particles, which may prevent the experimental observation of the energetically favorable cooperative wrapping of several particles in membrane tubes. Fine-tuning the adhesion energy in nanoparticle synthesis therefore may be essential to detect the cooperative wrapping of spherical particles in experiments. The cooperative wrapping should be easier to observe for prolate particles or the triblock Janus particles of figure 28(b) because the relatively high curvature at the tips of prolates and the non-adhesive caps of the Janus particles constitute a barrier for complete individual wrapping of these particles. The cooperative wrapping of spherical and prolate nanoparticles has been recently observed in CG simulations [158].

### Concluding remarks.

The interactions of nanoparticles and membranes affect the bioactivity of the particles because they need to be wrapped by membranes before entering the cells and cellular organelles of living organisms. Membrane-mediated interactions between adsorbed nanoparticles can lead to a cooperative wrapping of the nanoparticles and to linear assemblies in membrane tubes. These indirect interactions are related to the membrane-mediated interactions between embedded or adsorbed proteins, which can cause protein assembly and cooperativity. Advances in simulation techniques and nanoparticle synthesis may help to gain a deeper understanding of membrane-mediated interactions on molecular levels.

Acknowledgments

Financial support from the Deutsche Forschungsgemeinschaft (DFG) via the International Research Training Group 1524 ‘Self-Assembled Soft Matter Nano-Structures at Interfaces’ is gratefully acknowledged.
